# Three-dimensional mapping of hepatic lymphatic vessels and transcriptome profiling of lymphatic endothelial cells in healthy and diseased livers

**DOI:** 10.7150/thno.79953

**Published:** 2023-01-01

**Authors:** Songlin Huang, Borui Li, Zheng Liu, Mengli Xu, Dong Lin, Jiahong Hu, Dongjian Cao, Qi Pan, Jing Zhang, Jing Yuan, Qingming Luo, Zhihong Zhang

**Affiliations:** 1 Britton Chance Center and MoE Key Laboratory for Biomedical Photonics, Wuhan National Laboratory for Optoelectronics-Huazhong University of Science and Technology, Wuhan, Hubei 430074, China; 2 School of Biomedical Engineering, Hainan University, Haikou, Hainan 570228, China

**Keywords:** hepatic lymphatic vessels, *in vivo* fluorescence labeling and 3D imaging, hepatic lymphatic endothelial cell isolation, transcriptome sequencing, liver disease

## Abstract

**Rationale:** Hepatic lymphatics are essential for liver homeostasis and immune function. However, the 3D structure and spatial distribution of hepatic lymphatic vessels (LVs) need to be confirmed. Moreover, the molecular information of hepatic lymphatic endothelial cells (LyECs) needs to be further studied. The bottleneck is the lack of specific markers or labeling methods for hepatic lymphatic endothelial cells (LyECs)

**Methods:** Here, we proposed a method for the spatiotemporal sequential injection of antibodies (STSI-Ab) to selectively label hepatic LyECs *in vivo*. In addition, we also developed an efficient hepatic LyEC sorting method and performed deep transcriptome sequencing on hepatic LyECs.

**Results:** The STSI-Ab method achieved selective labeling of the mouse hepatic lymphatic network. Three-dimensional fluorescence imaging results of the STSI-Ab mouse liver lobe clearly showed that hepatic LVs entangled with the portal vein but were not present in the central vein. The imaging data inspired a novel hepatic lobule structure model with an added set of LVs in the portal area. Furthermore, deep transcriptome sequencing of isolated hepatic LyECs and Masson's trichrome staining results suggested that hepatic LyECs might be an important source of collagen fibers deposited in the portal area during the process of liver fibrosis and bile duct ligation (BDL).

**Conclusions:** We proposed an STSI-Ab method for selectively labeling hepatic LVs, distinguishing the hepatic LVs from other vessels, and mapping their 3D structure. This study opens an avenue for understanding hepatic lymphatic structure and it will be very beneficial to the study of hepatic LyEC functions.

## Introduction

The liver is a metabolic center and detoxification organ. In the past decade, the liver has also been considered a frontline immune tissue 1. It has been reported that the liver produces 25~50% of the total lymph fluid entering the thoracic duct 2. Thus, there are reasons to believe that the liver is a major site of lymphokinesis. However, research on hepatic lymphatics has been insufficient 3, 4. There is currently no complete atlas of hepatic lymphatics showing the fine structure of large LVs to lymphatic capillaries and their precise spatial positioning. For example, the French anatomist Rouvière proposed the structure and distribution of hepatic lymphatics at the macro level based on anatomical and computed tomography (CT) or magnetic resonance imaging (MRI) data. In this proposed model, the superficial lymphatic system is situated near the liver capsule, and the deep lymphatic system contains the periportal lymphatics and the perihepatic vein lymphatics 5, 6. In particular, hepatic lymphatic capillaries are an essential structural basis for draining excess fluids and proteins, the absorption of fats and fat-soluble vitamins, and the movement of immune cells between the liver and its draining lymph nodes. Hepatic lymphatics play an important role not only in the normal liver but also in diseased liver. Chronic liver diseases (CLDs), such as liver fibrosis, nonalcoholic fatty liver disease (NAFLD) and cholestasis, constitute a major medical and public health problem worldwide 7. Previous studies on CLDs mainly focused on blood vessels and liver cells, including hepatic sinusoidal endothelial cells and various immune cells, but little is known about the role of hepatic LyECs in CLDs. Therefore, exploring the intact 3D structure, the exact spatial localization of the hepatic lymphatics at the microscopic scale, and the function of hepatic LyECs in normal and diseased conditions is of great significance for thoroughly investigating the physiological function and pathological changes in the liver.

The key limiting factor for such investigations is the lack of a specific labeling method based on a unique marker for hepatic lymphatic endothelial cells (LyECs) to distinguish liver LVs from the extremely abundant blood vessels and bile ducts. The traditional specific marker for LyECs in other organs is lymphatic vessel endothelial hyaluronan receptor 1 (LYVE-1) 8. However, LYVE-1 is expressed in both hepatic LyECs and liver sinusoid endothelial cells (LSECs) 9. Although Prospero Homeobox 1 (Prox-1) 10 and podoplanin (PDPN) 11 are highly expressed in hepatic LyECs, they are also expressed in hepatocytes and cholangiocytes, respectively 3, 9, 12. Other molecular markers, such as vascular endothelial growth receptor 3 (VEGFR3) 13, chemokine (C-C motif) ligand 21 (CCL21), macrophage mannose receptor 1 (MMR1), desmoplakin and integrin α9, are expressed not only in hepatic LyECs but also in other cells (e.g., LSECs, Kupffer cells [KCs], cholangiocytes and hepatocytes) in the liver 3. Existing transgenic animals for labeling lymphatic vessels, such as Prox1-GFP 14, VEGFR3-tdTomato mice 15 and Mrc1a-eGFP zebrafish 16, could selectively label the lymphatic vessels in the heart, diaphragm, intestine and dermis. However, it is difficult to identify hepatic lymphatic vessels by using these transgenic animals, in which hepatocytes (Prox1^+^) or nearly half of the liver blood vessels (VEGFR-3^+^, Mrc1a^+^) were also marked. Since hepatocytes and blood vessels are present throughout the liver, the hepatic lymphatic vessels swamped in complex nonspecific signals. Therefore, it is urgent to develop a method to selectively label hepatic lymphatic vessels and clearly distinguish them from other vessels (blood vessels and bile ducts). However, it is currently difficult to use one unique marker to identify hepatic LyECs. The lack of both hepatic LyEC-specific markers and *in vivo* labeling methods hampers the investigation of the structure and function of hepatic lymphatics. Despite decades-long efforts, identifying a specific marker for hepatic LyECs has been understudied 17.

Considering the lack of specific markers for labeling hepatic LyECs, we expected that we could establish a method to selectively label LyECs in the whole liver, image their 3D structure and sort them for transcriptome sequencing. In previous works, researchers proposed that plasma components are filtered through the fenestrae of LSECs into the space of Disse to form the liver lymph, which then flows through the space of Mall and finally drains into LVs in the portal tract 18. It was reported that the liver lymph contained bilirubin after ligation of the common bile duct 19. In line with this finding, K. Yamamoto *et al*. found that resin injected into the common bile duct under pressure drains into the hepatic lymphatics 20. According to the differences in markers and the spatial positions of liver vessels, we proposed a spatiotemporal sequential injection of antibodies (STSI-Ab) method to selectively label hepatic LyECs. The intact liver LVs could be selectively labeled in two steps: first, intravenous (*i.v.*) injection of an antibody mixture to fluorescently label the liver blood vascular system and block the LYVE-1 molecule on LSECs; second, high-pressure injection of fluorescent anti-LYVE-1 antibody through the common bile duct into the bile flow in the retrograde direction at a high pressure. By using the STSI-Ab method, the intact hepatic lymphatics in the whole mouse liver were selectively labeled by fluorescent anti-LYVE-1 and were clearly identified in the environment of extremely abundant vessels by 3D fluorescence imaging. In addition, we proposed a method that can efficiently sort hepatic LyECs with high viability, and the cells are available for deep transcriptome sequencing. This research advances the understanding of the structure of hepatic lymphatics and the function of hepatic LyECs and will open up new possibilities for understanding hepatic lymphatics in liver physiology and pathology.

## Results

### The STSI-Ab method clearly distinguishes the lymphatics from blood vessels and bile ducts in the mouse liver

**Figure 1:**
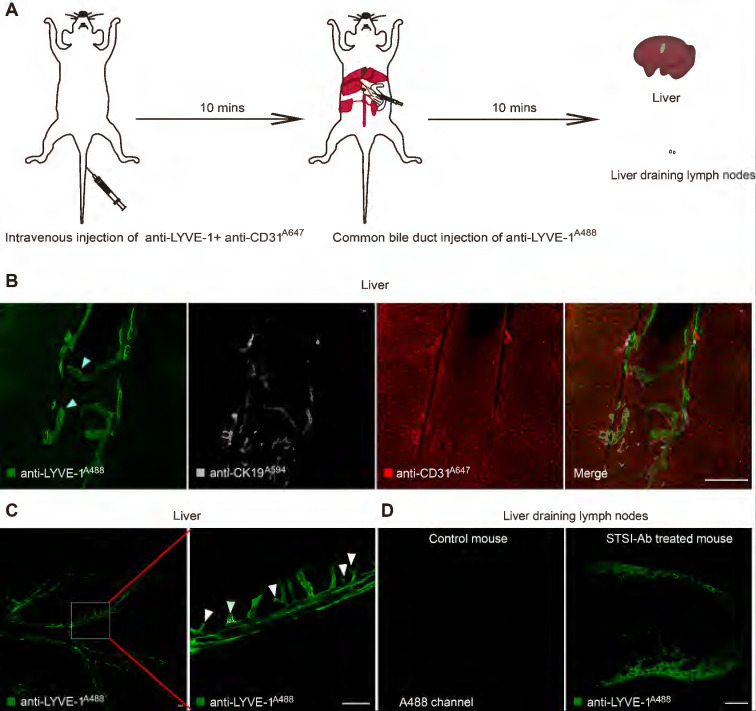
** STSI-Ab selectively labeled hepatic lymphatics.** (A) Schematic diagram of the STSI-Ab experimental procedure. (B) The green, gray and red vessels represent hepatic LVs, bile ducts and blood vessels, respectively (the blind ends of hepatic lymphatic capillaries are indicated by white arrowheads). (C) Panoramic view of the hepatic LVs. The imaging data clearly show the blind ends of hepatic lymphatic capillaries (indicated by white arrowheads). (D) STSI-Ab-labeled lymphatic sinuses in mouse liver draining lymph nodes (green, right). The control mouse was injected with PBS from the common bile duct, while no fluorescent signal was detected in the Alexa Fluor 488 channel (left panel D). LVs: lymphatic vessels. Scale bar: 200 µm.

To ensure that the liver LVs could be perfused by the fluid that was injected through the common bile duct at a high pressure, we first used Evans Blue to mark the vessels of the injection route. The results clearly showed that the liver-draining lymph nodes (LNs) (the portal and celiac LNs) 21, 22 and thoracic duct were rapidly stained blue within 10 s postinjection (Figure S1A-B). Although this method could quickly perfuse LVs in the whole liver, it did not achieve specific recognition of LVs due to the liver blood vessels and bile ducts also being stained. To solve this problem, utilizing the difference in the spatial positions of liver LVs and blood vessels, we proposed an STSI-Ab method for labeling, in which multiple antibodies were injected at two different sites at different time points. First, a mixture of an Alexa Fluor 647-conjugated anti-CD31 (anti-CD31^A647^) antibody (Table S1) and an anti-LYVE-1 antibody (without fluorescent dye to block LYVE-1 molecules on the surface of LSECs) was injected through the tail vein. Second, 10 min later, the Alexa Fluor 488-conjugated anti-LYVE-1 (anti-LYVE-1^A488^) antibody was injected through the common bile duct into the bile flow in the retrograde direction at a high pressure (Figure 1A). At this time, the hepatic LyECs in the LVs were labeled with an anti-LYVE-1^A488^ antibody, and the blood vessels were labeled with an anti-CD31^A647^ antibody. Finally, 10 min later, the mouse liver was obtained for tissue immunofluorescence imaging.

To obtain images with continuous vascular structures, we prepared 150-μm vibratome slices, which were subjected to immunostaining with a monoclonal rabbit anti-CK19 primary antibody to specifically label bile ducts, followed by incubation with an Alexa Fluor 594-conjugated goat anti-rabbit IgG secondary antibody (named primary and secondary antibodies as anti-CK19^A594^). Confocal imaging of liver tissue slices showed that three types of vessels were identified by anti-CD31^A647^ (blood vessels; red in Figure 1B), anti-LYVE-1^A488^ (LVs; green in Figure 1B, the blind ends of hepatic lymphatic capillaries are indicated by white arrowheads) and anti-CK19^A594^ (bile ducts; gray in Figure 1B). Thus, by using the STSI-Ab method, we successfully distinguished lymph vessels from blood vessels and bile ducts with very low cross-labeling among vessels. Only a few anti-CD31^A647^-labeled LSECs had extremely weak anti-LYVE-1^A488^ signals because the LYVE-1 molecules on LSECs were difficult to completely block by the *i.v.* injection of anti-LYVE-1.

Next, we performed large-field fluorescence imaging (3930 × 3930 μm) of thick tissue slices containing the most portal vein region (the left liver lobe was vibrated along the diaphragmatic surface to the visceral surface to approximately the 13^th^ layer, and each slice was 150 μm thick). The typical lymphatic vascular structures with blind ends (hepatic lymphatic capillaries) were only labeled with anti-LYVE-1^A488^ (Figure 1C white arrowheads). Confocal imaging of the liver draining lymph nodes showed that the lymphatic sinuses were also stained with anti-LYVE-1^A488^ (Figure 1D). In contrast to STSI-Ab, if the LYVE-1 antigens on LSECs were not blocked before anti-LYVE-1^A488^ injection, the injection of anti-LYVE-1^A488^ *via* the common bile duct would label both the hepatic LyECs and the LSECs (Figure S1C). In addition, the label of hepatic lymphatics by anti-LYVE-1^A488^ injection did not interfere with the *i.v.* injection of anti-CD31^A647^. Imaging data from 150-μm-thick tissue slices indicated that the diameter of liver LVs (11-63 μm, including lymphatic capillaries and large LVs in small and large portal veins) was larger than that of liver sinusoids (3.5-7.6 μm) (Figure S1C-D), and the mean fluorescence intensity (MFI) of anti-LYVE-1^A488^ on hepatic LyECs was higher than that on LSECs (Figure S1E).

In fact, we explored the appropriate blocking time because the anti-LYVE-1 antibody may pass through the liver sinusoids by *i.v*. injection and decreased the labeling efficiency of anti-LYVE-1^A488^ by blocking the LYVE-1 antigen on hepatic LyECs. We compared the MFI of anti-LYVE-1^A488^ on hepatic LyECs after 10 and 30 min of blocking (Figure S1F-G). In the imaging field of view, there was no anti-LYVE-1^A488^ fluorescence signal on LSECs at either time point (Figure S1F). As we detected a stronger anti-LYVE-1^A488^ fluorescence signal on hepatic LyECs at the 10-min time point than at the 30-min time point (Figure S1G), we chose to block the LYVE-1 molecules on LSECs for 10 min. Based on these results, we proposed an STSI-Ab method to selectively label LVs *in vivo*, providing an opportunity to map their structure.

### Anti-LYVE-1^A488^-labeled liver vessels expressed typical LyEC biomarkers after using the STSI-Ab method

**Figure 2:**
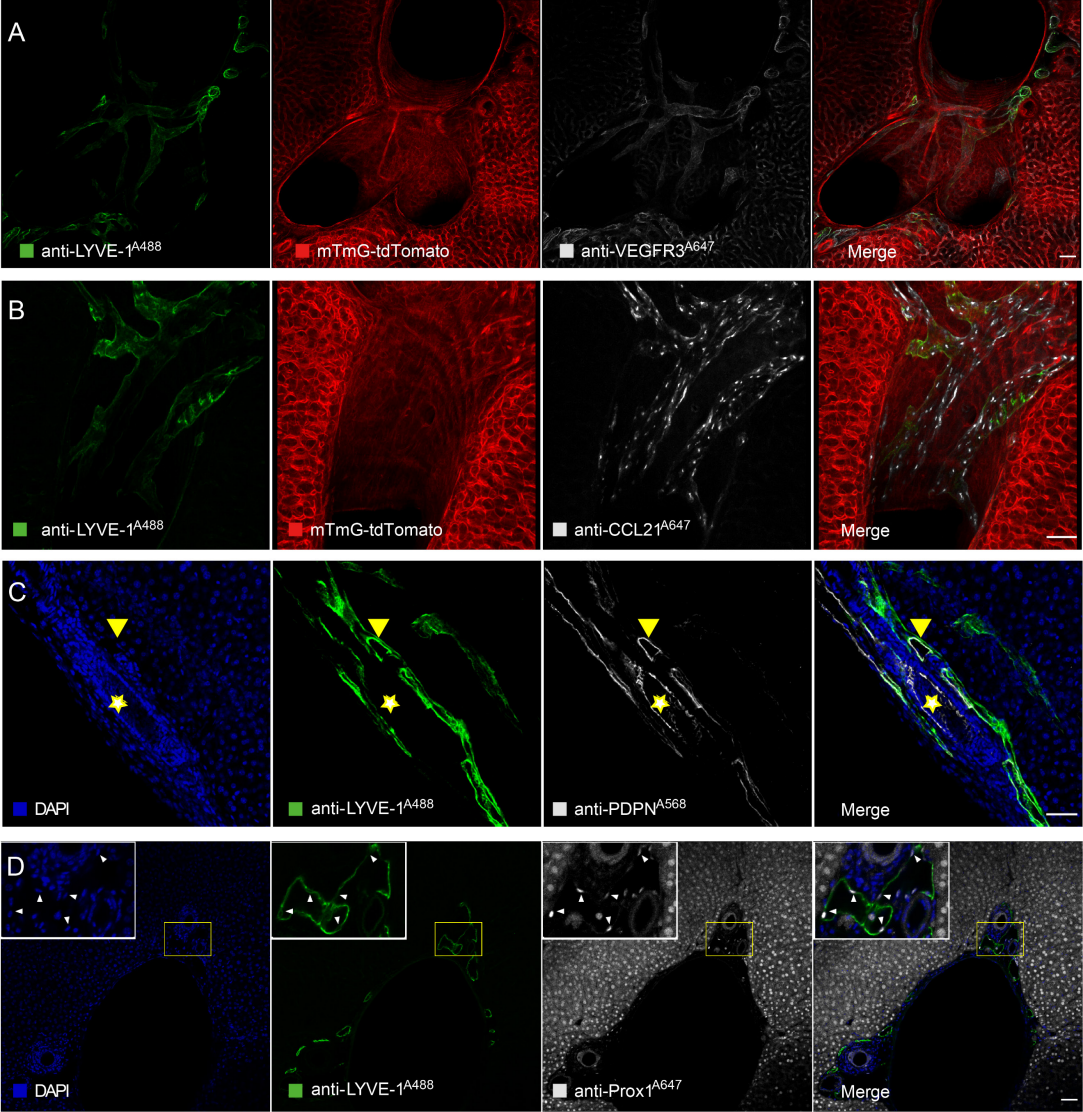
** Immunofluorescence imaging data confirmed that hepatic LyECs expressed multiple LyEC markers.** (A-B) Confocal imaging of liver slices of mTmG-tdTomato mice injected with anti-LYVE-1^A488^ *in vivo* using the STSI-Ab method showed that hepatic LyECs expressed VEGFR3 and CCL21 simultaneously. (C) The yellow arrowheads in panel C indicate a hepatic LV, and the yellow stars in panel C indicate a bile duct in a wild-type C57BL/6 mouse injected with anti-LYVE-1^A488^ *in vivo* using the STSI-Ab method. (D) Wild-type C57BL/6 mouse hepatic LyECs expressed Prox1 in the nucleus, and the hepatic LVs were labeled by the STSI-Ab method. The white arrowheads indicate that the LyEC nuclear marker Prox1 signals were colocalized with DAPI. LVs: lymphatic vessels. Scale bar: 50 µm.

To further confirm that anti-LYVE-1^A488^ in the STSI-Ab method selectively labels liver LVs *in vivo*, several common LyEC biomarkers were detected by immunofluorescence staining of liver tissue sections. Here, we used mTmG mice in Figure 2A and Figure 2B, in which tdTomato fluorescent protein was endogenously expressed on the cell membrane of most types of nucleated cells and was especially highly expressed on endothelial cells and cholangiocytes in the liver. When anti-LYVE-1^A488^ was injected through the common bile duct 10 min after *i.v.* injection of anti-LYVE-1, the hepatic lymphatics were labeled by anti-LYVE-1^A488^, while the liver blood vessels and bile ducts were revealed by tdTomato fluorescence, with no need for additional fluorescent antibody staining. The imaging data indicated that the anti-VEGFR3^A647^ signal colocalized with both anti-LYVE-1^A488^ and tdTomato fluorescence, indicating that lymphatic and blood vessels express similar levels of VEGFR3 (Figure 2A-B). Similarly, the anti-CCL21^A647^ signal mainly colocalized with anti-LYVE-1^A488^ and appeared as bright points (Figure 2B), indicating that CCL21 was highly expressed on LyECs but expressed at low levels on LSECs (Figure S2A). To further verify that anti-LYVE-1A488 in the STSI-Ab method selectively labels liver LVs, wild-type C57 mice were subjected to the STSI-Ab method (without *i.v.* anti-CD31^A647^ antibody), and the liver slices were then immunostained with anti-PDPN^A568^ and anti-Prox-1^A647^ antibodies (Figure 2C-D). Although PDPN is also expressed in cholangiocytes in the mouse liver and Prox-1 is also expressed in hepatocytes, we can distinguish the cell types according to the compact arrangement of cholangiocytes and the nuclear morphology of liver parenchymal cells. The imaging data showed that PDPN is expressed in both liver LVs (LYVE-1^+^PDPN^+^ vessels with loosely arranged nuclei, marked with yellow arrowheads in Figure 2C) and bile ducts (LYVE-1^-^PDPN^+^ vessels with closely arranged nuclei, marked with yellow stars in Figure 2C). As expected, both hepatic LyECs and parenchymal cells expressed Prox-1 in their nuclei (Figure 2D), which could be easily recognized according to their nuclear shape with a red bean-like shape in LyECs (indicated by white arrowheads) and a round shape in parenchymal cells. The imaging data clearly showed that the anti-LYVE-1^A488^ signal corresponded to Prox-1^+^ cells with red bean-shaped nuclei (indicated by the white arrowheads in Figure 2D). The quantitative data showed that the MFI of anti-VEGFR3^A647^, anti-PDPN^A568^ and anti-Prox-1^A647^ on hepatic LyECs was equal to that on LSECs, cholangiocytes and hepatocytes, respectively (Figure S2B-D). Taken together, these results showed that liver LVs coexpressed five LyEC biomarkers (LYVE-1^+^CCL21^+^VEGFR3^+^PDPN^+^Prox-1^+^ phenotype), which is different from the expression patterns of blood vessels (LYVE-1^+/-^CCL21^low^VEGFR3^+^PDPN^-^Prox-1^-^phenotype) and bile ducts (LYVE-1^-^CCL21^low^VEGFR3^-^PDPN^+^Prox-1^-^ phenotype). These results provided solid evidence that liver LVs were selectively labeled with LYVE-1^A488^ *via* the STSI-Ab method.

### Hepatic lymphatic vessels entangled with the portal vein

**Figure 3:**
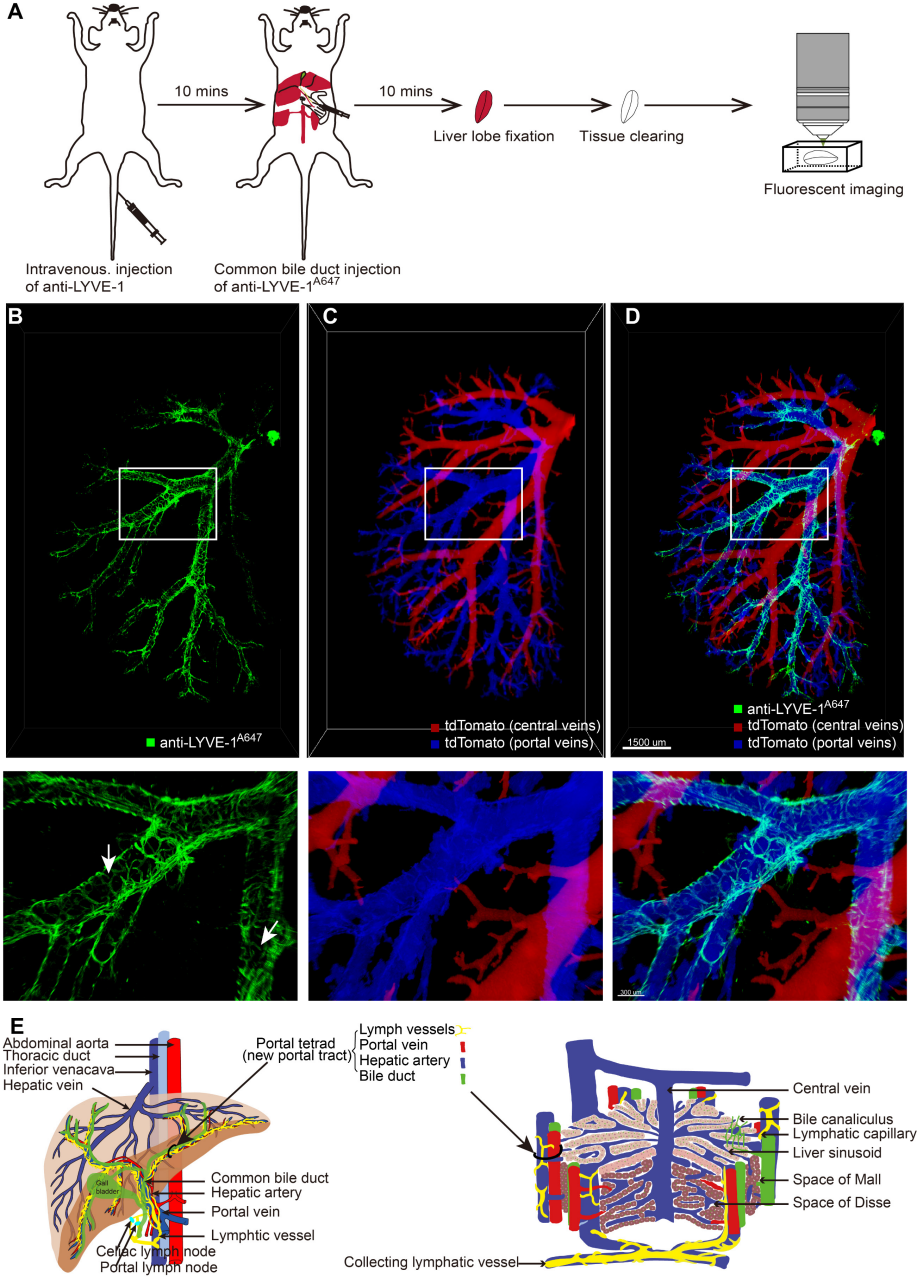
** Three-dimensional mapping of the mouse hepatic lymphatics.** (A) Mouse hepatic lymphatic 3D imaging flow diagram. (B-D) Green vessels represent mouse liver lymph vessels labeled with STSI-Ab, blue vessels represent the portal vein, and red vessels represent the central vein. The three images in the bottom row are the enlarged images in the white box in the top row. (E) Schematic diagram of liver vessels and hepatic lobule with portal tetrad.

After selectively labeling and confirming the identity of liver LVs, it was necessary to characterize their 3D structure and localization in the liver. Ten minutes after anti-LYVE-1 was *i.v.* injected, Alexa Fluor 647-conjugated anti-LYVE-1 (anti-LYVE-1^A647^) was injected through the common bile duct in the retrograde direction at a high pressure to label the hepatic lymphatics (Figure 3A). To obtain a clear 3D fluorescence image of intact liver LVs, the left liver lobe was subjected to FDISCO tissue optical clearing for 2 days 23 (Figure 3A). In this experiment, we also used mTmG mice to visualize the liver blood vessels and used the anti-LYVE-1^A647^ antibody to label liver LVs (Figure 3A) because the FDISCO optical clearing agents increased the imaging depth while enhancing the background fluorescence in the Alexa Fluor 488 channel. The 3D structure of the lymphatic vascular system was imaged using confocal microscopy with a 0.83×0.83×30 μm resolution. Liver blood vessels and LVs were clearly visible (Figure 3B-D). In general, the central vein and portal vein are larger in diameter than other vessels. In our 3D imaging data, the central vein (red vessels in Figure 3C) and portal vein (blue vessels in Figure 3C) can be distinguished by tracing the source of the vessel because the central vein is thicker than the portal vein. Hepatic LVs (green vessels in Figure 3B and 3D) entangled with the portal vein (Figure 3D, Movies S1), and there were no LVs at the central vein region (Figure 3D) or liver capsule region. The anti-LYVE-1^A647^-labeled hepatic LVs and the blind ends of lymphatic capillaries (white arrows in Figure 3B) distinguished LVs from blood vessels and bile ducts, even small blood capillary network did not have the blind-end structure 24. The 3D map of hepatic LVs displayed the continuous structure of the lymphatic capillaries to the large LVs (Figure S5A-B). Thus, the imaging results indicated that the liver has a powerful, well-designed lymphatic system to house and drain the lymph fluid produced in the liver. Moreover, the liver contains a large number of lymphatic capillaries (Figure 3B), which receive lymphatic fluid flow from sinusoids to lymphatic vessels in the portal areas. These numerous lymphatic capillaries provide a bridge for blood circulation and lymph circulation and play an important role in lymphatic drainage to maintain fluid homeostasis in the liver.

**Figure 4:**
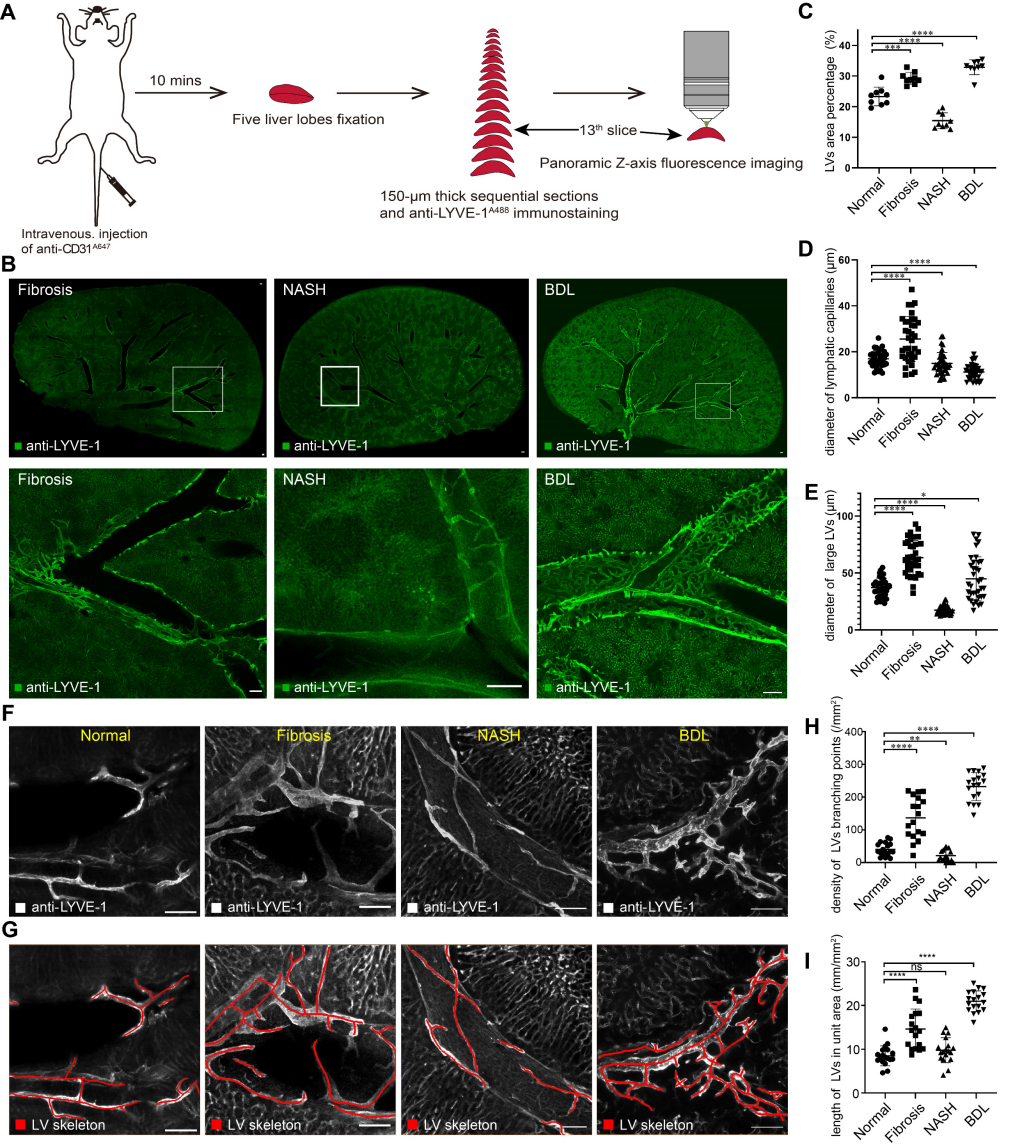
** Hepatic LV morphometric alterations in chronic liver diseases.** (A) Schematic diagram of liver slice immunostaining and imaging process. (B) Panoramic Z-stack of 150 µm liver slices after immunostaining with anti-LYVE-1^A488^ in CCl_4_-induced liver fibrosis, NASH and BDL model mice. The three images in the bottom row are the enlarged pictures in the white box in the top row. Scale bar: 200 µm. (C) Measurement of LV area percentage in normal and diseased livers (n = 3 mice in each group, 3 portal vein areas [including 2 large {300-400 µm} and 1 small {50-100 µm} portal vein area] were selected in each mouse). (D-E) Measurement of lymphatic capillary and large LV diameters in normal and diseased livers (n = 3 mice in each group, 4 portal vein areas [including 2 large {300-400 µm} and 2 small {50-100 µm} portal vein areas] were selected in each mouse, and 3 lymphatic capillaries and 3 large LVs were measured in each portal vein area). LVs, lymphatic vessels. (F-G) The skeletons of hepatic LVs (red lines) were drawn with Amira software. (H) Density of hepatic LV branching points in normal and diseased livers. (I) The length of hepatic LVs per unit area (six portal vein areas were randomly selected in each mouse, n = 3 mice in each group).

To verify that the hepatic lymphatic system existed only in the portal vein, the mice were *i.v.* injected with anti-CD31^A647^ antibody to label the blood system, and 10 min later, five liver lobes (named NO. 1-5 as shown in Figure S3A) were fixed and then prepared into 150-μm-thick vibratome slices. The slices were immunostained with anti-LYVE-1^A488^. Thus, CD31^-^LYVE-1^+^ vessels were hepatic LVs, while CD31^+^LYVE-1^+^ vessels were liver sinusoids. We screened the slices with the most LVs in every lobe under a fluorescence microscope for panoramic Z-axis fluorescence imaging (Figure 4A shows the preparation steps for the NO. 2 liver lobe). To save imaging time, we used homemade illumination modulation microscopy (Limo) 25, which covers the sample at 384 mm^2^/min with a pixel resolution of 0.325 μm × 0.325 μm, which is approximately 80 times faster than commercial point confocal microscopy. The results indicated that the spatial distribution of hepatic LVs labeled with this method is consistent with that of STSI-Ab, the hepatic lymphatic system existed only in the portal vein, and there were no LVs in the central vein or liver capsule (Figure S3B-E, Figure S4B). These results further proved that STATI-Ab can label hepatic lymphatics.

Our imaging results demonstrate that there are a set of "portal tetrads" in the portal area; that is, lymphatic vessels should be added to the “portal triad” (Figure 3E right). At the macroscopic liver level, the portal vein and hepatic artery carry nutrients and oxygen into the liver, respectively, while the bile ducts and lymphatic vessels drain bile and lymph fluid from the liver, respectively (Figure 3E left).

### Hepatic lymphatic alterations after chronic liver diseases

To understand the alterations of hepatic lymphatic morphological details in chronic liver diseases, we imaged hepatic LVs in CCl_4_-induced liver fibrosis, nonalcoholic steatohepatitis (NASH) and bile duct ligation (BDL) model mice with the same process in Figure 4A. The results of HE staining and Masson staining demonstrated the stability of the mouse models (Figure S4A). Imaging results indicated that in diseased mice, liver LVs still existed only in the portal area (Figure 4B, Figure S4C-E).

Additionally, we counted hepatic LV density under physiological and pathological conditions with Angio Tool software. The statistical results showed that the LV density (percentage of the area covered by LVs) 26 was 19.6-29.6% in normal livers. LV density was increased in fibrotic and BDL mice, and the densities were 26.6-32.9% and 27.0-35.4%, respectively. Conversely, hepatic LV density in NASH mice was reduced to 12.6-19.8% (Figure 4C). Similarly, we measured the diameters of lymphatic capillaries and large LVs in each group (Figure S5C). The results showed that the average diameter of hepatic lymphatic capillaries in normal mice was 17 μm, and after liver fibrosis, the average diameter of hepatic lymphatic capillaries increased to 25.6 μm. The average diameters of hepatic lymphatic capillaries in NASH and BDL mice were reduced to 15 μm and 11.2 μm, respectively (Figure 4D). The average diameter of large LVs in the liver fibrosis (63.5 μm) and BDL mice (44.9 μm) was enlarged compared with that in normal mice (37.2 μm), while the average diameter of the large LVs in the liver of NASH mice was reduced to 17.5 μm (Figure 4E). In addition, the skeletons of hepatic LVs (the red lines in Figure 4F-G) were drawn with Amira software to measure the density of hepatic LV branching points and the length of hepatic LVs in unit area. The results showed that the average density of hepatic LV branching points in normal mice was 40.5/mm^2^, and the average density of hepatic LV branching points was 136.9/mm^2^, 21.5/mm^2^, and 232.1/mm^2^ in CCl_4_-induced liver fibrosis, NASH and BDL mice, respectively (Figure 4H). The average length of hepatic LVs in each square millimeter was 8.5 mm in the normal mice and 14.6 mm, 9.8 mm and 21.0 mm in CCl_4_-induced liver fibrosis, NASH and BDL mice, respectively (Figure 4I).

Collectively, these results revealed hepatic LV morphometric alterations in chronic liver diseases. These results can help researchers better understand the structure of hepatic lymphatics in physiological and pathological conditions.

### Detaching the intact vascular architecture from the liver parenchyma for sorting hepatic LyECs and LSECs

Having proven that liver LVs entangled with the portal vein, we then assumed that the mouse liver could be digested by portal vein *in vivo* perfusion with moderate collagenase type IV. After digestion, the liver capsule was torn off, and the liver parenchyma was removed to obtain the liver vascular portion. At this point, the liver LVs remain in the vascular portion and maintain the intact structure. In this way, the coarse separation of liver LVs was achieved, and we can shorten the experimental time of hepatic LyEC sorting. Considering the impacts of flow cytometry sorting on cells, shortening the cell sorting time is beneficial to maintain cell viability, which is better for subsequent RNA sequencing experiments.

**Figure 5:**
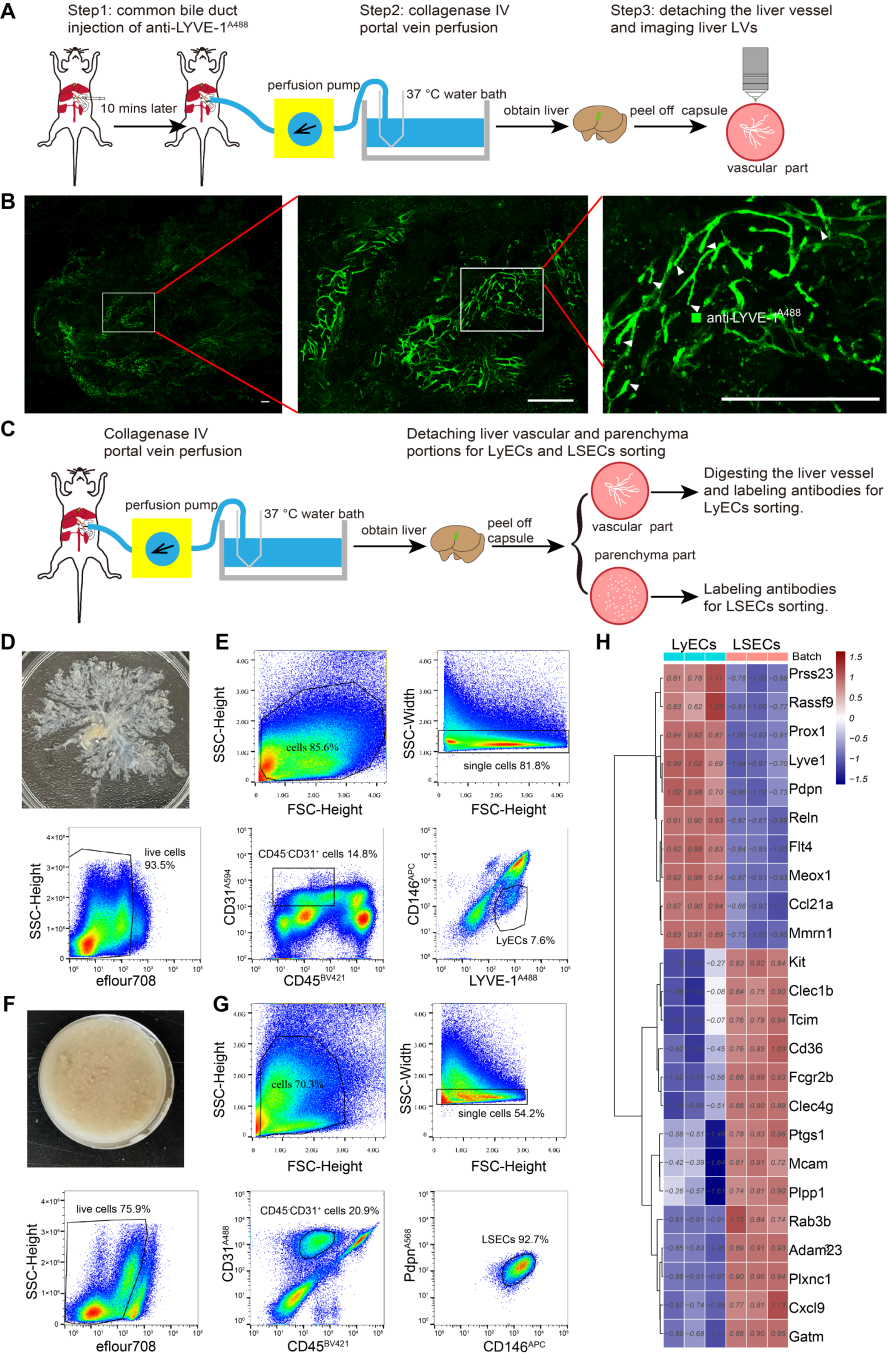
** Isolating hepatic LyECs and LSECs for deep transcriptome sequencing.** (A) Schematic diagram of the experimental procedure for hepatic vascular detachment and imaging. (B) Fluorescence imaging of hepatic LVs (green vessels). The white arrowheads indicate the blind ends (hepatic lymphatic capillaries). Scale bar: 500 µm. (C) Schematic diagram of the experimental procedure for hepatic LyEC sorting. (D) Image of the detached liver vascular portion. (E) The gating strategy for hepatic LyECs. (F) Picture of the liver parenchyma portion. (G) The gating strategy for hepatic LSECs. (H) Heatmap with hierarchical cluster analysis of marker genes. The marker genes were selected from the DEG set of normal hepatic LyECs compared with normal LSECs (n = 3 mice).

We further demonstrated this supposition by obtaining liver vascular portions through a 3-step procedure and performing fluorescence imaging of the liver vascular portion (Figure 5A-B). Step 1, *in vivo* labeling of hepatic LVs: anti-LYVE-1^A488^ was injected into the common bile duct to label the whole-hepatic lymphatics and LYVE-1^+^ LSECs *in vivo*. Step 2, collagenase IV portal vein perfusion: collagenase digestion caused the liver vessels to detach from the liver parenchyma, and the hepatic sinusoid, parenchymal cells, and liver capsule were separated. The liver sample was separated into the liver vascular portion and the parenchyma portion, which were used to sort LyECs and LSECs, respectively. Step 3, confocal imaging: the imaging data confirmed that the vascular portion maintained well-structured liver vessels (Figure 5B green vessels), and the magnified images also showed hepatic lymphatic capillaries (white arrowheads in Figure 5B).

For LyEC sorting, the mice were treated with portal vein perfusion of collagenase IV solution to detach the liver vascular and parenchyma portions without prior antibody injection (Figure 5C). In this process, we did not use the STSI-Ab method because we had to avoid the overlap of fluorescent molecules when using multiple antibodies in cytometry sorting. The detached liver vascular portions (Figure 5D) were digested into single-cell suspensions, and then the single-cell suspensions of the vascular portions were labeled with BV421-conjugated anti-CD45 antibody (anti-CD45^BV421^), anti-LYVE-1^A488^, Alexa Flour 594-conjugated anti-CD31 antibody (anti-CD31^A594^), and APC-conjugated anti-CD146 antibody (anti-CD146^APC^). The hepatic LyECs were gated by CD45^-^CD31^+^LYVE-1^+^CD146^-^, and the fixable viability dye eFlour780 was used to exclude dead cells (Figure 5E).

The single-cell suspensions of the parenchyma portions (Figure 5F) were labeled with anti-CD45^BV421^, monoclonal rat anti-mouse CD31 primary antibody and an Alexa Fluor 488 conjugated goat anti-rat IgG secondary antibody (named primary and secondary antibodies as anti-CD31^A488^), anti-CD146^APC^, monoclonal hamster anti-PDPN primary antibody and an Alexa Fluor 568-conjugated donkey anti-hamster IgG secondary antibody (named primary and secondary antibodies as anti-PDPN^A568^). The LSECs were gated by CD45^-^CD31^+^CD146^+^PDPN^-^ (Figure 5G).

To compare the molecular phenotypes of hepatic LyECs and LSECs, we sorted hepatic LyECs and LSECs for deep transcriptome sequencing. The established markers of LyECs (Lyve-1, Prox1, Pdpn, Ccl21a, Mmrn1, Reln, and Rassf9) are highly expressed in hepatic LyECs. In contrast to LyECs, Mcam, Fcgr2b (CD32b), Cxcl9, CD36, Kit, Clec4g and other genes were highly expressed in LSECs (Figure 5H). The sequencing data further proved the accuracy of these two types of cells.

In summary, we provide a reliable method for detaching liver vessels from the intact mouse liver and precisely sorting hepatic LyECs. It is convenient to analyze the transcriptome status of hepatic LyECs under physiological and pathological conditions.

### Transcriptome sequencing compared the gene expression of hepatic LyECs and LSECs in normal and diseased livers

**Figure 6:**
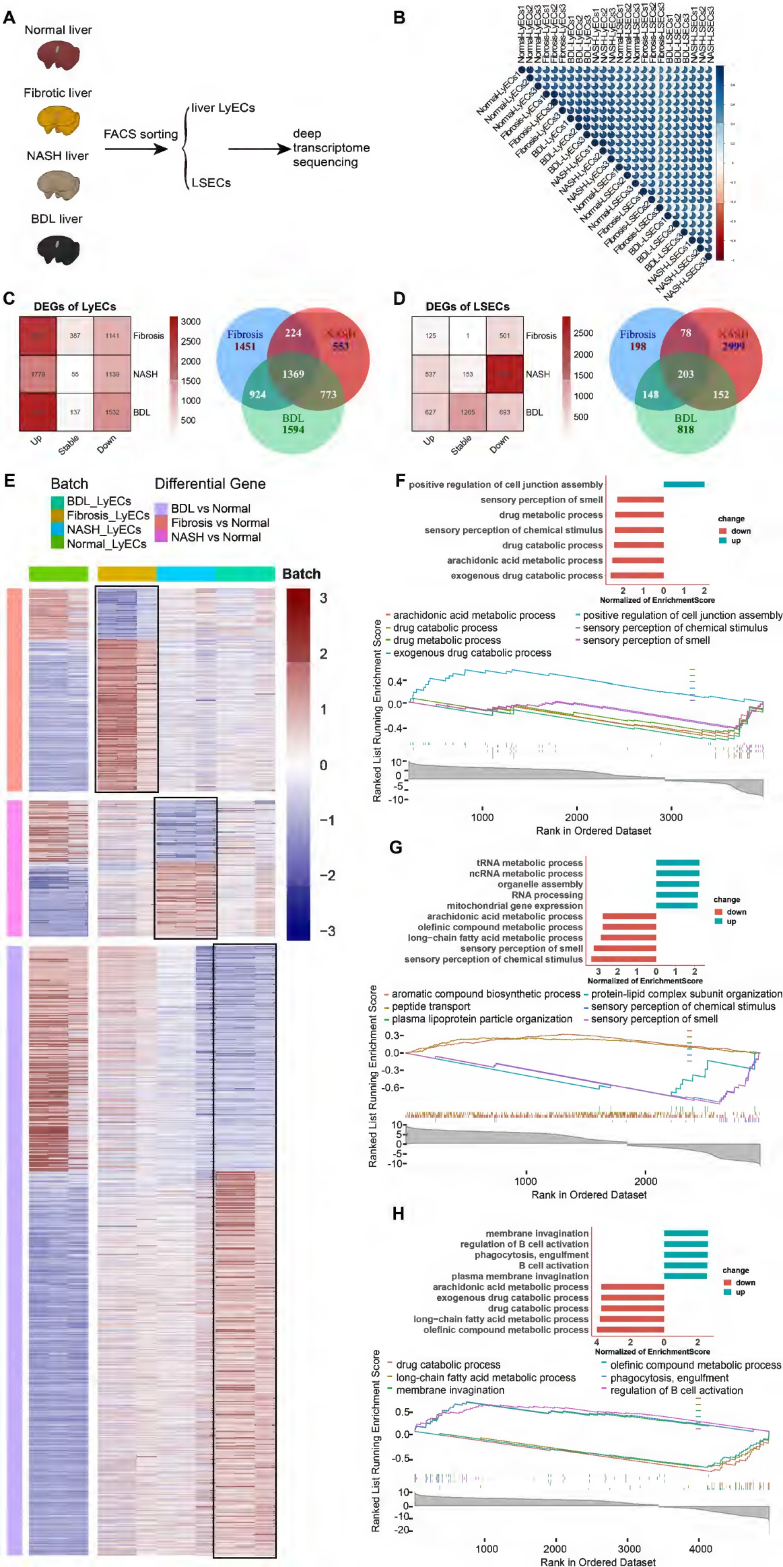
** Transcriptome sequencing analysis and comparison of hepatic LyECs between normal and diseased mice.** (A) Schematic diagram of the experimental process. (B) The correlation between 6 groups of LyECs and LSECs (n = 3 mice). (C) Venn diagram of the overlapping DEGs in hepatic LyECs from the normal, liver fibrosis, nonalcoholic steatohepatitis and bile duct ligation groups (n = 3 mice). (D) Venn diagram of the overlapping DEGs in LSECs from the normal, liver fibrosis, nonalcoholic steatohepatitis and bile duct ligation groups (n = 3 mice). (E) Heatmap shows DEGs in hepatic LyECs between normal and three disease model mice. (F) Gene set enrichment analysis (GSEA) for normal hepatic LyECs and fibrotic hepatic LyECs using GO terms. (G) Gene set enrichment analysis (GSEA) for normal LyECs and nonalcoholic steatohepatitis hepatic LyECs using GO terms. (H) Gene set enrichment analysis (GSEA) for normal LyECs and bile duct ligation hepatic LyECs using GO terms. A P value < 0.05 and adjusted P value < 0.05 were considered statistically significant; GO, Gene Ontology; NES, normalized enrichment score.

To explore the transcriptome profiling of hepatic LyECs and LSECs in liver diseases, we sorted hepatic LyECs and LSECs from normal, CCl_4_-induced liver fibrosis, NASH and BDL mice for deep transcriptome sequencing (Figure 6A). Stringent quality control (QC) and screening were used for processing raw data. Sequencing generated a total of eight datasets, consisting of three replicate samples in each dataset (Table S2). We pairwise compared the Pearson correlation of three replicates in each dataset, and the intragroup correlation coefficient was greater than the between-group correlation coefficient (Figure 6B). The established markers of LyECs (Prox1, Pdpn, Ccl21a, Mmrn1, Reln, and Rassf9) are highly expressed in hepatic LyECs from CCl_4_-induced liver fibrosis, NASH and BDL mice. In contrast to LyECs, Mcam, Fcgr2b (CD32b), Cxcl9, CD36, Kit, Clec4g, etc., genes were highly expressed in LSECs from CCl_4_-induced liver fibrosis, NASH and BDL mice (Figure S6A). The sequencing data further proved the accuracy of these cells. There was reason to believe that the results obtained by sequencing were reliable, which reflected the stability of the sorting method mentioned above.

Furthermore, we performed a quantitative comparison of the DEGs between the normal group and the above disease model groups (Figure 6C-D). Compared with the normal groups, there were 3967 (2826 upregulated and 1141 downregulated genes) differentially expressed genes (DEGs) in fibrotic hepatic LyECs, 2918 (1779 upregulated and 1139 downregulated genes) DEGs in NASH hepatic LyECs, and 4660 (3128 upregulated and 1532 downregulated genes) DEGs in BDL hepatic LyECs. Among these three sets of DEGs, there were 1369 common genes, which means that these genes were prominently expressed in all situations. We identified unique DEGs in the three groups (1451 genes in fibrotic group, 553 genes in NASH group, and 1594 genes in BDL). The unique genes showed the features of different groups (Figure 6C, Table S3). Then, we showed the DEGs in hepatic LyECs using a heatmap (Figure 6E). Our transcriptome sequencing results showed decreased Prox1 expression in the LyECs of liver fibrosis mice (Figure S6B). We also observed a decrease in the expression of the tight junction proteins ZO-1 (Tjp1) and Esam in LyECs after liver fibrosis (Figure S6B), which indicated that the permeability of hepatic LyECs was affected. In addition, Gene Set Enrichment Analysis (GSEA, Table S4) showed that the drug metabolic and exogenous drug catabolic processes of hepatic LyECs in CCl_4_-induced liver fibrosis mice were downregulated (Figure 6F). Unexpectedly, GSEA showed that cell junction assembly of hepatic LyECs was upregulated after liver fibrosis (Figure 6F), and tight junction protein Claudin-3 (Cldn3) expression was upregulated (Figure S6B). These results suggested that in the process of liver fibrosis, the permeability of lymphatic vessels was not simply increased by downregulating the expression of Prox1, ZO-1, Esam, etc., but also by upregulating the expression of Claudin-3, etc., to increase the cell junction assembly of hepatic LyECs, implying that the stability of hepatic LVs might reach a balance in positive and negative regulation of tight junction proteins.

In NASH hepatic LyECs, the upregulated biological processes were mostly enriched in RNA metabolic processes and organelle assembly, while the downregulated biological processes mainly included sensory perception and fatty acid metabolism (Figure 6G). In BDL hepatic LyECs, the processes related to B-cell activation and phagocytosis were upregulated, while the downregulated biological processes were mostly enriched in arachidonic acid metabolic processes and exogenous drug catabolic processes (Figure 6H). These results suggested that the exogenous catabolic function of hepatic LyECs was reduced in these three liver diseases. The liver is an immunologically tolerant organ 27, while in BDL, hepatic LyECs might enhance the humoral immune response by activating B cells, suggesting that hepatic LyECs might play a role in promoting the liver immune response in certain pathological conditions.

Fibrotic deposits are observed in chronic liver diseases. Our transcriptome sequencing results showed that extracellular matrix genes, such as Col1a1, Col1a2, Col3a1, Col5a1, Col6a1, Col6a2, Col6a3, and Col14a1, were upregulated in hepatic LyECs of liver fibrotic mice, and Col5a1 and Col6a3 were upregulated in hepatic LyECs of BDL mice. In contrast, the expression of Col1a1, Col1a2, Col3a1, Col4a5, Col6a1, Col6a2, Col8a1, Col14a1, Col15a1 and Fbn1 in hepatic LyECs was decreased during NASH compared with normal conditions (Figure S6B). The Masson staining results showed collagen deposition in the portal areas of CCl_4_-induced liver fibrosis and BDL mice but not in NASH mice (Figure S4A). These results suggested that hepatic LyECs may be an important source of collagen fibers deposited in the portal area during the process of liver fibrosis and BDL.

The same method was used to analyze LSECs from the normal group and three disease-model groups. There were 626 (125 upregulated and 501 downregulated genes) differentially expressed genes (DEGs) in the LSECs of liver fibrosis mice, 3432 (537 upregulated and 2895 downregulated genes) DEGs in the LSECs of NASH mice and 1320 (627 upregulated and 693 downregulated genes) DEGs in the LSECs of BDL mice. In LSECs, there were only 203 common DEGs, while there were 2999 unique genes in the NASH group compared with the normal group (Figure 6D). Then, we showed the DEGs in LSECs using a heatmap (Figure S7A). GSEA showed functional changes in the LSECs of NASH mice, and the upregulated biological processes were associated with macroautophagy and blood vessel endothelial cell migration, whereas the downregulated biological processes mainly included nervous system processes, sensory perception and the G protein-coupled receptor signaling pathway (Figure S7C). In CCl_4_-induced liver fibrosis mice, the upregulated biological processes of LSECs were associated with blood circulation and circulatory system processes, but the biological processes associated with leukocyte activation and immune response were downregulated (Figure S7B). In LSECs of BDL mice, the biological processes associated with sensory perception of chemical stimulus were upregulated, while immune system processes were downregulated (Figure S7D).

### Hepatic LyECs have a unique transcriptomic signature compared with LyECs in other organs

**Figure 7:**
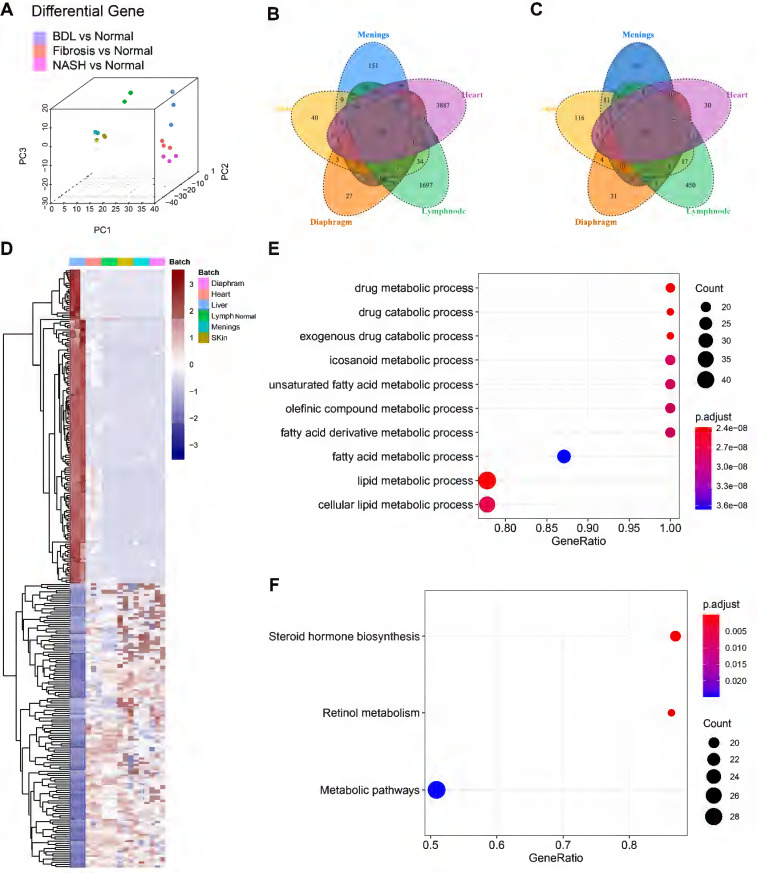
** Transcriptome sequencing analysis and comparison of LyECs between mouse liver and other organs.** (A) 3D-principal component analysis (PCA) was used to show the data after batch effect processing. (B-C) The Venn diagram of DEGs in LyECs from the liver and other organs, fold change > 2, P value < 0.05 in B; the fold change < 2^-1^, P value < 0.05 in C (n = 3 mice). (D) Heatmap with hierarchical cluster analysis of DEGs in LyECs from the liver and other organs. (E) Gene set enrichment analysis (GSEA) for hepatic LyECs compared in other organs using GO terms. (F) Gene set enrichment analysis (GSEA) for hepatic LyECs compared in other organs using the KEGG database. A P value < 0.05 was considered statistically significant; GO, Gene Ontology; KEGG, Kyoto Encyclopedia of Genes and Genomes.

To investigate whether LyECs in the liver differ from those in other tissues, we compared the transcriptomic characteristics of hepatic LyECs to those of LyECs from other tissues. We collected datasets of different mouse organs from published papers, such as the meninges, diaphragm, skin 28 [GSE99743], heart 29 [GSE150041] and lymph nodes 30 [GSE119499]. We obtained raw data from the GEO database and performed quality control and data filtering. We use the "ComBat" algorithm 31 to remove the batch effect on all datasets, making the multiple organ data comparable. After batch processing, the integrated data are shown in the PCA plot (Figure 7A). After a unified pipeline of mapping and counting, the data we collected will be used for subsequent analysis. To reduce the impact of biological experiments and sequencing technology on the data, the diverse data were integrated after correcting for confounding factors and batch effects. The integrated data are shown in the PCA plot. The proportions of PC1, PC2 and PC3 variance were 53.65%, 7.04% and 5.52%, respectively (Figure 7A, Table S5). Compared with LyECs of other organs, there were 153 upregulated genes and 159 downregulated genes in hepatic LyECs (Figure 7B-C). The common upregulated or downregulated genes (Figure 7D, Table S6) were used for functional enrichment studies to analyze the unique transcriptomic significance of the liver (Table S7). GSEA showed the differences among the LyECs of the liver and other organs using GO terms, and almost all results were enriched for metabolism-related biological processes (Figure 7E). To further explore the importance of liver-specific genes, GSEA using the KEGG database can focus on precise pathways. We found that the steroid hormone biosynthesis pathway and the retinol metabolism pathway were enriched (Figure 7F). Such an expression profile suggested that the hepatic LyECs may be involved in steroid hormone biosynthesis functions of the liver.

## Discussion

In this study, to reveal the spatial distribution and 3D structure of hepatic lymphatics, we proposed an STSI-Ab method for selectively labeling hepatic LyECs* in vivo* and distinguishing hepatic lymphatics from blood vessels and bile ducts in the whole liver. The 3D imaging results provided high-resolution structural mapping of liver LVs, which clearly showed the continuous structure of the lymphatic capillaries to the large LVs (Figure 3B, Figure S5A-B), thereby identifying hepatic LVs in the environment of extremely abundant vessels. Previous studies suggested that there are three sets of LVs in the human liver that are located in the liver capsule, portal vein and central vein 6. Our results showed an abundant set of LVs in the mouse liver wrapping around the portal vein. We also performed panoramic imaging of all mouse liver lobes but did not detect LVs around the central vein and liver capsule (Figure S3 and Figure S4). Although there may be species differences, it is certain that there is a powerful lymphatic system entangled with the portal vein. Based on these results, we propose modifying the model hepatic lobule, the basic structure and the functional unit of the liver. The model should have a set of LVs coexisting in the portal area with the portal vein, hepatic artery and bile duct (Figure 3E).

Previous studies used two-dimensional imaging to analyze hepatic LV diameters, and the results suggested that hepatic LVs expand in chronic liver diseases 32, 33. In this study, the Z-axis imaging results of 150-μm-thick vibratome sections presented the tubular structure of hepatic LVs, and then the LV density, diameters, density of LV branching points and length of hepatic LVs in unit area were measured (Figure 4, Figure S5C). The results indicated that LV density was increased in liver fibrotic and BDL mice but decreased in NASH mice (Figure 4C). The diameter of hepatic lymphatic capillaries increased after liver fibrosis but decreased in NASH and BDL mice (Figure 4D). The diameter of large LVs in the liver fibrotic and BDL mice was enlarged compared with that in normal mice but contracted in the NASH mice (Figure 4E). In addition, the average density of hepatic LV branching points and the average length of hepatic LVs in each square millimeter were increased in liver fibrotic and BDL mice (Figure 4H-I), while the average density of hepatic LV branching points was decreased in NASH mice (Figure 4H). Thus, the Z-axis imaging results allowed for comprehensive assessment of vascular alterations, and the quantitative data revealed significant changes in LV density, diameters of large LVs and lymphatic capillaries, and LV branching and length during the progression of CCl_4_-induced liver fibrosis, NASH and BDL.

It should be noted that the segmentation and measurement of hepatic LVs are challenging due to the irregular structure of LV capillaries. These processes are mainly operated manually and are time-consuming. One limitation of this study is that the number of samples in this article is relatively small. In the future, the development of image segmentation and automatic calculation methods for multilevel LVs with different sizes will contribute to accurately and efficiently obtaining LV information from panoramic Z-stack imaging of the liver lobe (or thick section) in normal and diseased conditions. On the other hand, in the STSI-Ab method, 150 μl of PBS-containing anti-LYVE-1 antibody was injected from the common bile duct in the retrograde direction to bile flow in 3 s. Since the anti-LYVE-1 antibody is smaller than 10 nm in size and the gaps of discontinuous button-like junction structures on lymphatic capillaries are larger than 100 nm 34, no obvious structural damage was observed in the 3D continuous structure of the lymphatic capillaries (indicated by white arrowheads in Figure S5) to the large LVs (indicated by white dotted lines in Figure S5).

To date, there have been a few reports on liver single-cell transcriptome sequencing with clustered hepatic LyECs 32, 33, 35. However, data for deep sequencing analysis of hepatic LyECs have not been reported. At present, the only reported method for sorting mouse hepatic LyECs is to use CD45^-^CD31^+^CD146^-/low^PDPN^+^ to define hepatic LyECs 33, and the sorting rate of hepatic LyECs in whole liver cells is very low (< 0.01%). In our method, after detaching the liver vascular portion, the proportion of hepatic LyECs was higher than that in the whole liver; the proportion of hepatic LyECs in viable cells was 1.13% (14.8%×7.64%) (Figure 5E), and the sorting time of hepatic LyECs was shortened compared with the current hepatic LyEC sorting method 33. Considering the effects of flow cytometry sorting on cells, shortening the cell sorting time is beneficial to maintain cell viability, which is better for subsequent RNA sequencing experiments.

Hepatic LVs may play an important role in pathological processes such as liver fibrosis and liver cancer 29. Our sequencing results indicated that hepatic LyECs and LSECs undergo different changes during the process of liver fibrosis (Figure 6, Figure S6 and Figure S7). Previous studies have shown that oxidized low-density lipoprotein (oxLDL) impacts lymphatic stability *via* a reduction in Prox1 expression and impedes LyEC metabolism 32, 33. Our transcriptome sequencing results also showed decreased Prox1 expression in LyECs from liver fibrosis mice, and the expression of the tight junction proteins ZO-1 (Tjp1) and Esam in LyECs was downregulated after liver fibrosis (Figure S6B), which indicated that the permeability of hepatic LyECs was affected. However, GSEA showed that cell junction assembly of hepatic LyECs was upregulated after liver fibrosis (Figure 6F), and tight junction protein Claudin-3 (Cldn3) expression was upregulated (Figure S6B). The results suggested that in the process of hepatic fibrosis, the stability of liver LVs might reach a balance in the positive and negative regulation of tight junction proteins.

It is generally believed that in the process of liver fibrosis, the extracellular matrix of the liver is mainly derived from hepatic stellate cells. After liver fibrosis, there will be fiber deposition around the liver sinusoids, and the fiber scars in the portal area are more obvious. Therefore, collagen fibers deposited in the portal area may originate from other types of cells. The deep transcriptome sequencing results showed that many extracellular matrix genes were upregulated in hepatic LyECs in liver fibrotic and BDL mice, while many extracellular matrix genes in hepatic LyECs were decreased during NASH (Figure S6B). The Masson staining results also supported that collagen deposition in the portal areas of CCl_4_-induced fibrotic mice or BDL mice was higher than that of normal mice. There was no significant difference between the NASH and normal mice (Figure S4A). Considering that hepatic LVs existed in the portal area (Figure 3D), these results together suggested that hepatic LyECs may be an important source of collagen fibers deposited in the portal area during the process of liver fibrosis and BDL. This study may provide a new target cell (hepatic LyECs) for reversing the liver fibrosis process.

## Conclusion

In conclusion, we proposed an STSI-Ab method for selectively labeling liver LVs, distinguishing the liver LVs from other vessels, and mapping their 3D structure. This study revealed previously unknown intact hepatic lymphatics from lymphatic capillaries to large lymphatic vessels (LVs) with definite spatial coordinates, which provided a reference for clinical liver lymphatic angiography. Furthermore, we established a new method for accurately sorting hepatic LyECs for transcriptome sequencing. Our data suggested that hepatic LyECs may be an important source of collagen fibers deposited in the portal area during the process of liver fibrosis and BDL, which would be considered a potential target for reversing the liver fibrosis process.

## Materials and Methods

### Animals

Female mTmG (stock NO. 007676) mice were purchased from Jackson Laboratories, and female C57BL/6 mice were purchased from Changsha Hunan Silaike Jingda Laboratory Animal Co., Ltd. (Changsha, Hunan, China). Mice were housed and bred at the Animal Center of Wuhan National Laboratory for Optoelectronics for at least 1 week before the experiments and were used in studies when 6-8 weeks old. All animal studies were conducted according to protocols that had been approved by the Hubei Provincial Animal Care and Use Committee and in accordance with the experimental guidelines of the Animal Experimentation Ethics Committee of Huazhong University of Science and Technology.

### CCl_4_-induced liver fibrosis

Adult mice (6-8 weeks old) were used to establish the CCl_4_-induced liver fibrosis model. Mice were administered a CCl_4_ 1:3 mixture with corn oil (2.5 ml/kg, *i.p.*) twice weekly for eight weeks.

### Mouse model of NASH

The methionine-choline deficient (MCD) diet was used for a mouse nonalcoholic steatohepatitis (NASH) model. Six- to eight-week-old mice were fed the MCD diet (10 g of diet per 20 g body weight) for 3 weeks, and the diet was repeated every two days.

### Mouse model of BDL

A diet containing 0.1% 3,5-diethoxycarbonyl-1,4-dihydrocollidine (DDC) was used for the mouse model of cholestatic liver disease. Six- to eight-week-old mice were fed a 0.1% DDC diet (10 g of diet per 20 g body weight) for 2 weeks, and the diet was repeated every two days.

### Bile common duct injection of Evans Blue to stain liver draining lymph nodes

Step 1 The mice were euthanized by *i.p.* injection of a mixture of 10 mg/kg xylazine and 100 mg/kg ketamine hydrochloride (Sigma, St. Louis, Missouri, USA).

Step 2 The skin and abdominal muscle were split and moved to the mid-axillary line to expose the liver, and the intestine was poked with a cotton swab to expose the common bile duct.

Step 3 A total of 150 μl of 1 mg/ml Evans Blue was injected from the common bile duct in the retrograde direction to bile flow in 3 s. Then, the liver draining lymph nodes were stained with Evans Blue in 10 s.

### STSI-Ab method

Step 1 Adult mice were *i.v.* injection of 200 μl of PBS containing 30 µg anti-LYVE-1 and 15 µg anti-CD31^A647^ antibodies.

Step 2 Ten minutes later, mice were euthanized by *i.p.* injection of a mixture of 10 mg/kg xylazine and 100 mg/kg ketamine hydrochloride (Sigma, St. Louis, Missouri, USA).

Step 3 The skin and abdominal muscle were split and moved to the mid-axillary line to expose the liver, and the intestine was poked with a cotton swab to expose the common bile duct.

Step 4 A total of 150 μl PBS containing 30 µg anti-LYVE-1^A488^ antibody was injected from the common bile duct in the retrograde direction to the bile flow in 3 s.

Step 5 Ten minutes later, the mice were transcardially perfused with 0.1 M PBS, and then the liver and its draining lymph nodes were harvested and fixed in 4% (w/v) PFA (pH 7.4) in PBS at 4°C and subjected to follow-up experiments.

### Tissue immunofluorescence staining

Step 1 The mouse liver lobes and lymph nodes were fixed in 4% PFA for 10 h at 4°C.

Step 2 The liver lobes were prepared into 150-μm thick slices by using a vibrating blade microtome (Leica VT1200, Germany). The lymph nodes were dehydrated in 25% (w/v) sucrose solution for 10 h at 4°C and then prepared into 15-μm thick slices using a cryostat (Leica CM1950, Germany).

Step 3 Liver and lymph node slices were incubated with PBS containing 1% BSA and 0.2% Triton-X-100 for 1 h at room temperature (RT).

Step 4 The slices were incubated with appropriate dilutions of primary antibodies for 24 h at 4°C in PBS containing 1% BSA and 0.2% Triton-X-100.

Step 5 The slices were washed 3 times for 5 min at RT with PBS, followed by incubation with secondary antibodies for 1 h at RT.

Step 6 The slices were washed 3 times for 5 min at RT with PBS, followed by incubation with 5 µg/ml DAPI reagent (Sigma‒Aldrich) for 20 min at RT.

Step 7 The slices were washed with PBS, mounted with glass slides under coverslips and imaged with an LSM 710 confocal laser scanning microscope (Zeiss, Germany).

To verify the selective labeling ability of the STSI-Ab method of LVs, the livers in STSI-Ab-labeled mice were harvested and incubated with the rabbit polyclonal anti-CK19 primary antibody (Abcam, 1:800; specifically labeling bile ducts) and the secondary antibody Alexa Fluor 568-conjugated goat anti-rabbit IgG antibody (Invitrogen, 1:2000). To demonstrate that multiple LyEC markers were expressed in hepatic LyECs, the primary antibodies used for tissue immunofluorescence were as follows: goat anti-VEGFR3 (R&D Systems, 1:800), goat anti-CCL21 (R&D Systems, 1:800), Syrian hamster anti-podoplanin (Abcam, 1:800), and rabbit anti-Prox1 (Abcam, 1:800). The secondary antibodies corresponding to the above primary antibodies were Alexa Fluor 647-conjugated donkey anti-goat IgG antibody (Invitrogen, 1:2000), Alexa Fluor 568-conjugated goat anti-Syrian hamster IgG antibody (Abcam, 1:2000) and Alexa Fluor 647-conjugated goat anti-rabbit IgG antibody (Invitrogen, 1:2000). All slices were imaged with an LSM710 laser confocal scanning microscope (Zeiss, Germany). The data were analyzed using ImageJ software.

### Panoramic Z-stack imaging of liver lobe serial vibratome slices

After fixation of the liver lobes, all slices of serial vibratome slices of intact liver lobes (single sheet 150 µm) were subjected to immunostaining. Then, the required slices from each liver lobe were chosen, and panoramic Z-stack imaging was performed using homemade illumination modulation microscopy (Limo).

### Three-dimensional imaging of mouse hepatic lymphatics

Step 1 Adult mice were *i.v.* injection of 200 μl of PBS containing 30 µg anti-LYVE-1 antibody.

Step 2 Ten minutes later, the mice were euthanized by *i.p.* injection of a mixture of 10 mg/kg xylazine and 100 mg/kg ketamine hydrochloride (Sigma, St. Louis, Missouri, USA).

Step 3 The skin and abdominal muscle were split and moved to the mid-axillary line to expose the liver, and the intestine was poked with a cotton swab to expose the common bile duct.

Step 4 A total of 150 μl of PBS containing 30 µg anti-LYVE-1^A647^ antibody was injected from the common bile duct in the retrograde direction into the bile flow in 3 s.

Step 5 The mice were transcardially perfused with 0.1 M PBS, and the livers were harvested. The liver lobes were separated and fixed in 4% (w/v) PFA (pH 7.4) at 4°C for 10 h.

Step 6 The fixed liver lobes were washed three times (30 min each time) in PBS at room temperature.

Step 7 The liver lobes were dehydrated through gradient tert-butanol (30%, 50%, 70%, 80%, 90% and 96% diluted in H_2_O) for 6 h for each concentration at 4°C.

Step 8 BABB-D10 (108014, Sigma‒Aldrich) was used as a refractive index matching solution to clear the tissue after dehydration for 2 h at room temperature.

Step 9 The cleared liver lobes were removed and subjected to fluorescence imaging with an LSM710 laser confocal scanning microscope (Zeiss, Germany).

### Hepatic lymphatic and blood vessel reconstruction

The imaging data were processed with Imaris software. Briefly, the imaging signal was first reversed by the “Invert Channel(s)” module to highlight the large vessel in the liver. The 3D structure of the highlighted vessel was then reconstructed through the “Surfaces” module. The portal vein and the hepatic vein were differentiated in “Surfaces” by the diameter difference. Finally, differentiated vessels were filled with different colors after 3D reconstruction through the “Mask Channel” step in the “Surfaces” module.

### Detaching liver vessels for fluorescence imaging

Step 1 The adult mice were euthanized by *i.p.* injection of a mixture of 10 mg/kg xylazine and 100 mg/kg ketamine hydrochloride (Sigma, St. Louis, Missouri, USA).

Step 2 The skin and abdominal muscle were split and moved to the mid-axillary line to expose the liver, and the intestine was poked with a cotton swab to expose the common bile duct.

Step 3 A total of 150 μl PBS containing 5 µg anti-LYVE-1^A488^ antibody was injected from the common bile duct in the retrograde direction into the bile flow in 3 s.

Step 4 Ten minutes later, the mice were perfused with 0.1 M PBS for 2 min *via* the portal vein using a perfusion pump at 35 rpm.

Step 5 Low-glucose DMEM containing 0.5 mg/ml collagenase IV (37°C water bath) was perfused *via* the portal vein for 20 min using a perfusion pump at 9 rpm.

Step 6 The mouse livers were carefully excised, and the gallbladders were removed. Then, the livers were placed into Petri dishes containing 2 ml of DMEM.

Step 7 We carefully peeled off the liver capsule with sharp tweezers. Then, liver parenchymal cells were carefully knocked off using the inner core of the insulin syringe.

Step 8 Thus, the liver vascular portions were successfully detached and mounted with glass slides under coverslips and imaged with an LSM 710 confocal laser scanning microscope (Zeiss, Germany).

### Detaching liver vessels for hepatic LyEC and LSEC isolation

Step 1 The adult mice were euthanized by *i.p.* injection of a mixture of 10 mg/kg xylazine and 100 mg/kg ketamine hydrochloride (Sigma, St. Louis, Missouri, USA).

Step 2 The skin and abdominal muscle were split and moved to the mid-axillary line to expose the liver, and the intestine was poked with a cotton swab to expose the common bile duct.

Step 3 The mice were perfused with 0.1 M PBS for 2 min *via* the portal vein using a perfusion pump at 35 rpm.

Step 4 Low glucose DMEM containing 0.5 mg/ml collagenase IV (37°C water bath) was perfused *via* the portal vein for 20 min using a perfusion pump at 9 rpm.

Step 5 The mouse livers were carefully excised, and the gallbladders were removed. Then, the livers were placed into Petri dishes containing 2 ml of DMEM.

Step 6 We carefully peeled off the liver capsule with sharp tweezers. Then, liver parenchymal cells were carefully knocked off using the inner core of the insulin syringe. The liver parenchymal portions were placed in new Petri dishes for LSEC isolation.

Step 7 The liver vascular portions were cut into pieces with scissors and placed in 1 ml of collagenase IV perfusion digestion solution containing 0.5 mg of DNase I, 4 mg of neutral protease, and 2 mg of DNase I in a 37°C water bath for 5 min. The cells from the liver vascular portions were used for hepatic LyEC isolation.

Step 8 The single-cell suspensions of liver parenchymal and liver vascular portions were pipetted with a liquidizer and filtered with 200 mesh gauze.

Step 9 Then, the suspensions were centrifuged at 400×g for 4 min at 4°C, the supernatants were discarded, and the cell pellets were resuspended with 1 mmol/L EDTA in PBS.

Step 10 The suspensions were incubated with the Fixable Viability Dye eFluor 780 (eBioscience, 1:1000) for 30 min at 4°C and then washed.

Step 11 The suspensions were incubated with Fc antibody for 20 min at 4°C and then washed.

Step 12 The single-cell suspension of the vascular portions was labeled with anti-CD45^BV421^, anti-LYVE-1^AF488^, anti-CD31^AF594^, and anti-CD146^APC^ for 30 min at 4°C and then washed twice.

Step 13 The single-cell suspension of the parenchyma portions was labeled with anti-CD45^BV421^, anti-CD31^A488^, anti-CD146^APC^, and anti-PDPN^A568^ for 30 mins at 4°C and then washed twice.

Step-14 Single-cell suspensions of liver vascular and liver parenchymal portions were used for hepatic LyEC and LSEC isolation, respectively.

All antibodies used in this study are shown in Table S1.

### RNA sequencing, Quality Control and Alignment of Sequencing Data

For RNA sequencing, all processing and bulk sequencing were performed according to a streamlined RNAseq (EASY RNAseq) protocol 36 by Biowavelet Ltd, Chongqing, China. EASY RNAseq can obtain transcriptome information from small numbers of cells.

The raw sequencing reads in fastq format were processed with FastQC (v0.11.8). To reduce data noise and ensure the accuracy and reliability of downstream analysis results, it is necessary to filter unqualified sequence reads. Data cleaning and filtering were analyzed using Trimmomatic (v0.39) software. After filtering, the remaining reads were called Clean Reads and stored in fastq format for downstream analysis processes.

After quality control (QC), the clean sequencing reads underwent two stages of processing to obtain the raw counts. First, the reads were mapped to the Mus musculus genome (mm39) using HISAT software (2.1.0-4). Then, the number of reads aligning to each gene was quantified with HTSeq (0.13.5). The Hmisc(4.7.0) R package was used to examine the correlation between samples.

### Data integration

To compare the lymphatic endothelial cells of different organs, we used the same pipeline to process lymphatic endothelial cells from different tissues. To eliminate the batch effect brought by biological experiments and sequencing technologies, we used the function “ComBat-seq” in the sva (3.35.2) R package to integrate the data. 3D visualization of PCA was used to display the integrated data with the “scatterplot3d” (0.3.41) R-package.

### Differentially Expressed Genes (DEGs)

We used the Negative Binomial (Also known as Gamma-Poisson) distribution approach of the DESeq2 (1.30.1) R-package to obtain DEGs. The |log2(fold change) | > 1, false discovery rate (FDR) < 0.05 and P-value < 0.05 are regarded as the cutoff to screen for differentially expressed genes (DEGs).

### Venn diagram and Heatmap

A Venn diagram was used to describe common and unique DEGs in different groups. To compare the differences in genes in different groups, we showed the differentially expressed genes in each group using a Heatmap. Venn diagram was generated by the VennDiagram (1.6.20) R-package and ggplot2 (3.3.5) R-package. We used the GenomicFeatures(1.42.3) R-package to obtain Fragments Per Kilobase of exon model per Million mapped fragments (FPKM ). After Logarithmization and standardization, we used the Pheatmap (1.0.12) R-package to make the Heatmap with row clustered by Pearson correlation.

### Gene Set Enrichment Analysis (GSEA)

The differentially expressed genes (DEGs) were enriched in GO terms (GO database: http://geneontology.org). The gene set of GO term enrichment analysis and visualization between samples were performed by R package clusterProfiler (v3.16.1). A P value < 0.05 was considered significant.

### Statistical analyses

Student's t test for independent samples was applied for the evaluation of LV diameters and space, LyEC ratio, and the MFI of different fluorescence antibodies. We counted hepatic LV density under physiological and pathological conditions with Angio Tool software. To count hepatic LV density, we randomly selected 3 portal vein areas (including 2 large [300-400 µm] and 1 small [50-100 µm] portal vein areas) in the panoramic image of 150-μm-thick liver vibratome slices from each mouse (n = 3 mice). We then measured hepatic LV density (percentage of the area covered by vessels) with Angio Tool software. To compare hepatic lymphatic diameters in normal, CCl_4_-induced liver fibrosis, NASH and BDL mice, we measured the diameters of lymphatic capillaries and large LVs in each group consisting of 3 mice. The five liver lobes of each mouse were cut into 150-μm-thick serial vibratome sections. For each mouse, 150-μm-thick vibratome sections with the most LVs were selected from each liver lobe. Then, the 150-μm-thick vibratome sections were subjected to panoramic Z-stack imaging. That is, each group has 15 panoramic Z-stack imaging data of 150-μm thick vibratome sections. Subsequently, 2 large (diameter 300-400 μm) and 2 small (diameter 50-100 μm) portal vein regions were randomly selected from the 15 panoramic Z-stack images, and then 3 lymphatic capillaries and 3 large LVs were selected from each region to measure their diameters. This means that the diameters of 36 lymphatic capillaries and 36 large LVs were measured for each group. All data were represented and evaluated using GraphPad Prism 8.0 software, and the acceptable levels of significance were set at *P* < 0.05. The flow cytometry data were analyzed using FlowJo v7.6.3 software.

## Supplementary Material

Supplementary figures and table 1, table legends.

Supplementary tables 2-7.

Supplementary movie.
