# Supplementary material for: Three-dimensional mapping of hepatic lymphatic vessels and transcriptome profiling of lymphatic endothelial cells in healthy and diseased livers
Source: Theranostics. 2023 Jan 1;13(2):639–58. doi: 10.7150/thno.79953 (PMC9830445; doi:10.7150/thno.79953)
Supplement: Supplementary file 1 — Supplementary figures and table 1, table legends. [file thnov13p0639s1.pdf]

Supporting Information

Three-dimensional mapping of hepatic lymphatic vessels and transcriptome profiling of lymphatic endothelial cells in healthy and diseased livers

Songlin Huang,[1]† Borui Li,[1]† Zheng Liu,[1]† Mengli Xu [1], Dong Lin[1], Jiahong Hu[1], Dongjian Cao[1], Qi Pan[1], Jing Zhang[1], Jing Yuan[1], Qingming Luo[2,]*, Zhihong Zhang[1,2]*

[1] Britton Chance Center and MoE Key Laboratory for Biomedical Photonics, Wuhan National Laboratory for Optoelectronics-Huazhong University of Science and Technology, Wuhan, Hubei 430074, China

[2] School of Biomedical Engineering, Hainan University, Haikou, Hainan 570228, China

† These authors contributed equally: Songlin Huang, Borui Li, Zheng Liu

*Correspondence: Zhihong Zhang, czyzzh@mail.hust.edu.cn;

Qingming Luo, qluo@hainanu.edu.cn

Address: Room G304, Britton Chance Center for Biomedical Photonics, Wuhan National Laboratory for Optoelectronics–Huazhong University of Science and Technology, Wuhan 430074, China. Fax: +86-27-87792034; Tel: +86-27-87792033;

Zhihong Zhang (czyzzh@mail.hust.edu.cn) is the corresponding author to communicate with the Editorial and Production offices.

Supplementary Materials

This part includes:

Figure S1 Intravenous injection of anti-LYVE-1 blocked LYVE-1 molecules on LSECs but not on hepatic LyECs.

Figure S2. Quantitative comparison of the expression of four LyEC markers between hepatic LyECs and other cells.

Figure S3. Confocal imaging of LV distribution in liver lobes in mice.

Figure S4. Confocal imaging of LV and blood vessel distribution in normal and diseased mice.

Figure S5. Three-dimensional mapping of hepatic LVs displayed the continuous structure of the lymphatic capillaries to the large LVs.

Figure S6. Transcriptome sequencing analysis and comparison of LyECs and LSECs between normal and diseased livers.

Figure S7. Transcriptome sequencing analysis and comparison of LSECs between normal and diseased livers.

Table S1. Catalog of antibodies used in this study.

Table S2. FPKM values of hepatic LyECs and LSECs from normal and diseased livers.

Table S3. DEGs of hepatic LyECs and LSECs from normal and diseased livers.

Table S4. GO and KEGG analysis of the DEGs of hepatic LyECs and LSECs from the normal and diseased livers.

Table S5. FPKM of LyECs in the liver and other organs.

Table S6. DEGs of LyECs in the liver and other organs.

Table S7. GO and KEGG analysis of the DEGs of LyECs in the liver and other organs.

Movie S1 Three-dimensional mapping of the hepatic lymphatics and blood vessels. The central vein is shown in red; the portal vein is shown in blue; and the hepatic lymphatic system is shown in green.

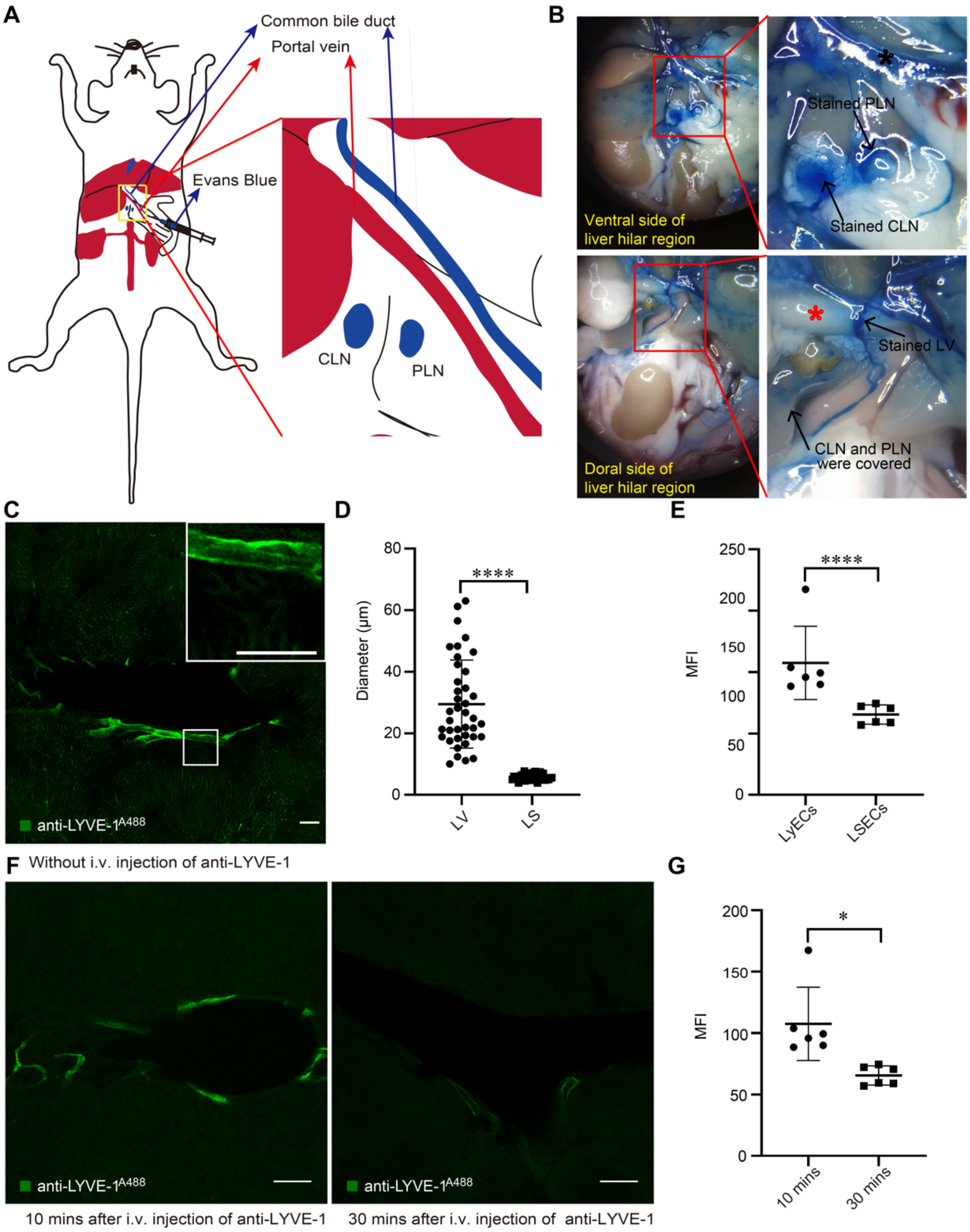

Figure S1 Intravenous injection of anti-LYVE-1 blocked LYVE-1 molecules on LSECs but not on hepatic LyECs. (A-B) After injection of Evans Blue through the common bile duct at a high pressure, mouse liver draining lymph nodes in the hilar area were stained by Evans Blue. CLN, celiac lymph node; PLN, portal lymph node; LV, lymphatic vessel. The black asterisk indicates the common bile duct, and the red asterisk indicates the esophagus. (C) Confocal imaging of the mouse liver slices from the mouse without *i.v.* injection of anti-LYVE-1 in advance. The imaging data showed that both the hepatic LyECs and the LSECs were labeled by anti-LYVE-1^{A488}. (D) The

quantitative data of the diameters of hepatic LVs (n = 39 LVs consisting of lymphatic capillaries and large LVs in 3 mice per group) and liver sinusoids (LS) (n = 39 liver sinusoids in 3 mice per group). (E) Quantitative fluorescence data of the anti-LYVE-1^{A488} signal in hepatic LyECs and LSECs (n = 39 vessels in 3 mice per group). (F) Confocal imaging of mouse liver slices from mice injected with the anti-LYVE-1^{A488} from the common bile duct 10 minutes (left) and 30 minutes (right) after *i.v.* injection of anti-LYVE-1. (G) Quantitative fluorescence data of the anti-LYVE-1^{A488} signal in F. MFI: mean fluorescence intensity (n = 6 LVs per group). Scale bar: 100 µm.

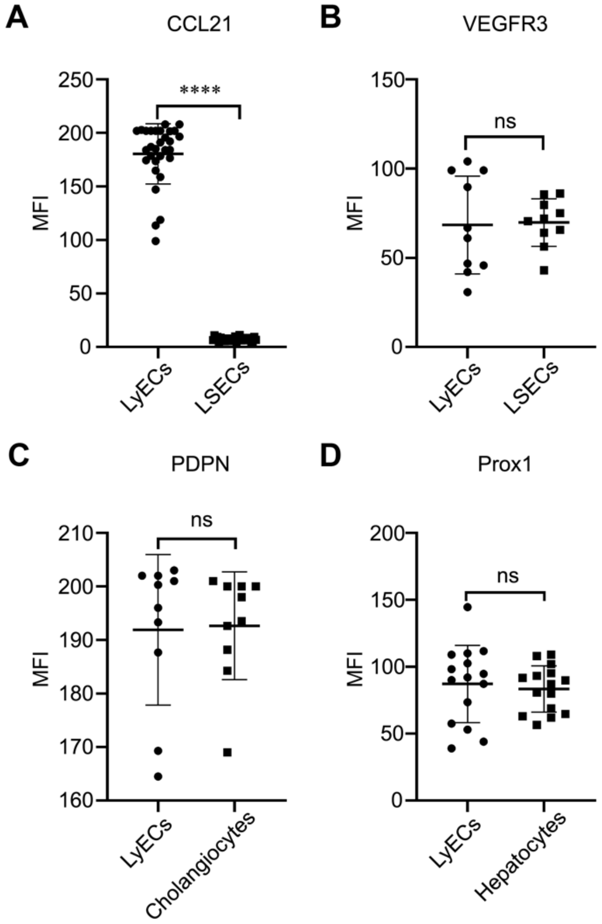

Figure S2. Quantitative comparison of the expression of four LyEC markers between hepatic LyECs and other cells. (A-B) Quantitative fluorescence data of anti-CCL21^{A647} and anti-VEGFR3^{A647} on hepatic LyECs and LSECs, respectively. (C) Quantitative fluorescence data of anti-PDPN^{A568} on hepatic LyECs and cholangiocytes. (D) Quantitative fluorescence data of anti-Prox1^{A647} on hepatic LyECs and hepatocytes, n = 3 mice.

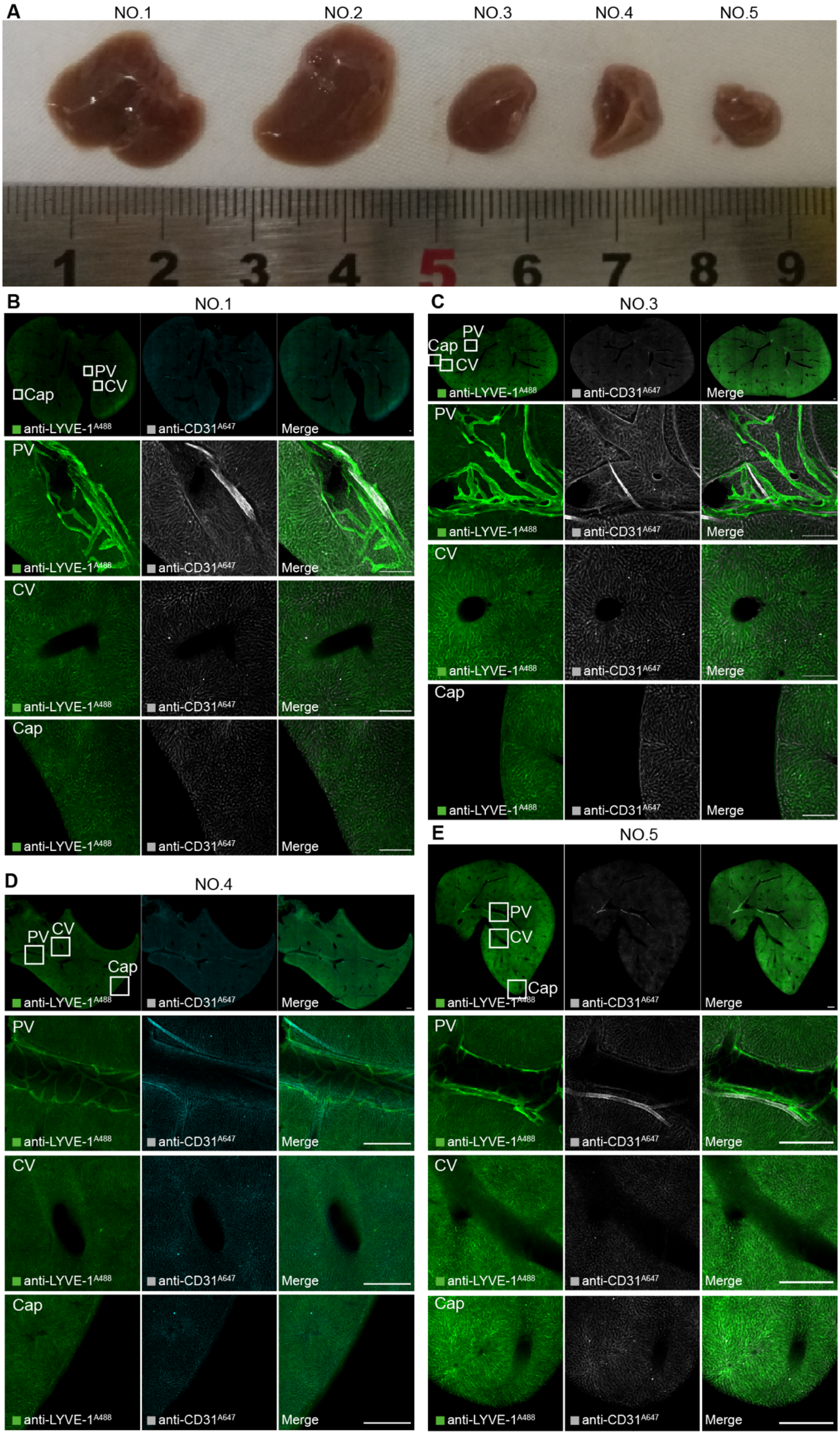

A
NO.1
NO.2
NO.3
NO.4
NO.5
1 2 3 4 5 6 7 8 9
B
NO.1
Cap
PV
CV
anti-LYVE-1A488
anti-CD31A647
Merge
C
NO.3
D
NO.4
E
NO.5

Figure S3. Confocal imaging of LV distribution in liver lobes in mice. (A) Images of five liver lobes labeled NO. 1-5. (B-F) Representative panoramic Z-stack images of LVs and blood vessels in the NO. 1, 3, 4 and 5 liver lobes. PV: portal vein, CV: central vein, cap: liver capsule. LV, lymphatic vessel. Scale bar: 200 μm.

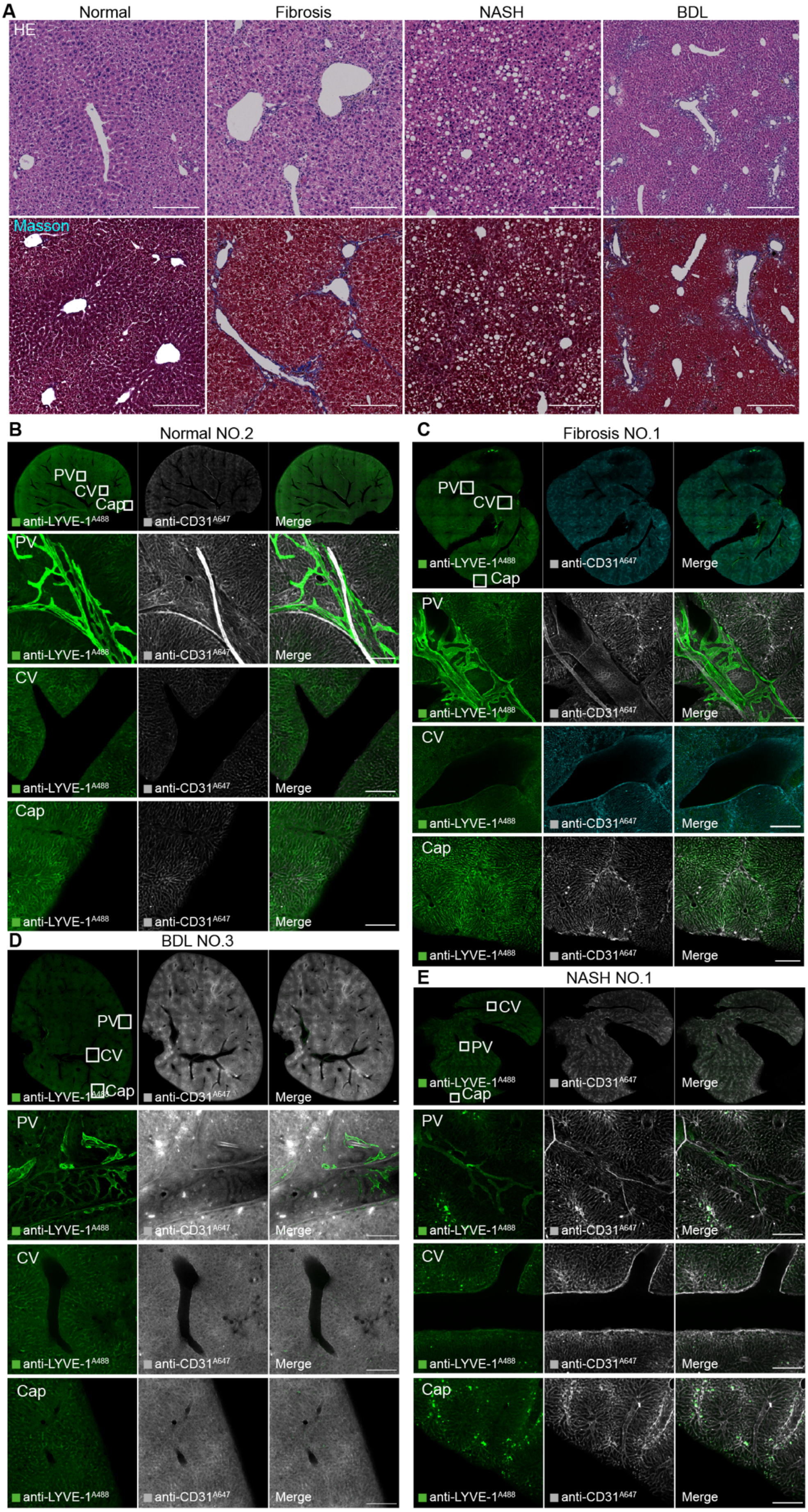
A
Normal
Fibrosis
NASH
BDL
HE
Masson
B
Normal NO.2
PV
CV
Cap
anti-LYVE-1A488
anti-CD31A647
Merge
C
Fibrosis NO.1
D
BDL NO.3
E
NASH NO.1

Figure S4. Confocal imaging of LV and blood vessel distribution in normal and diseased mice. (A) HE and Masson imaging of the liver in normal, CCl_4-induced liver fibrosis, NASH and BDL model mice. (B) Representative panoramic Z-stack images of LVs and blood vessels in the NO. 2 liver lobe in normal mice. (C) Representative panoramic Z-stack images of LVs and blood vessels in the NO. 1 liver lobe in CCl_4-induced liver fibrosis mice. (D) Representative panoramic Z-stack images of LVs and blood vessels in the NO. 3 liver lobe in bile duct ligation mice. (E) Representative panoramic Z-stack images of LVs and blood vessels in the NO. 1 liver lobe in nonalcoholic steatohepatitis mice. PV: portal vein, CV: central vein, cap: liver capsule, LVs, lymphatic vessels. Scale bar: 200 μm.

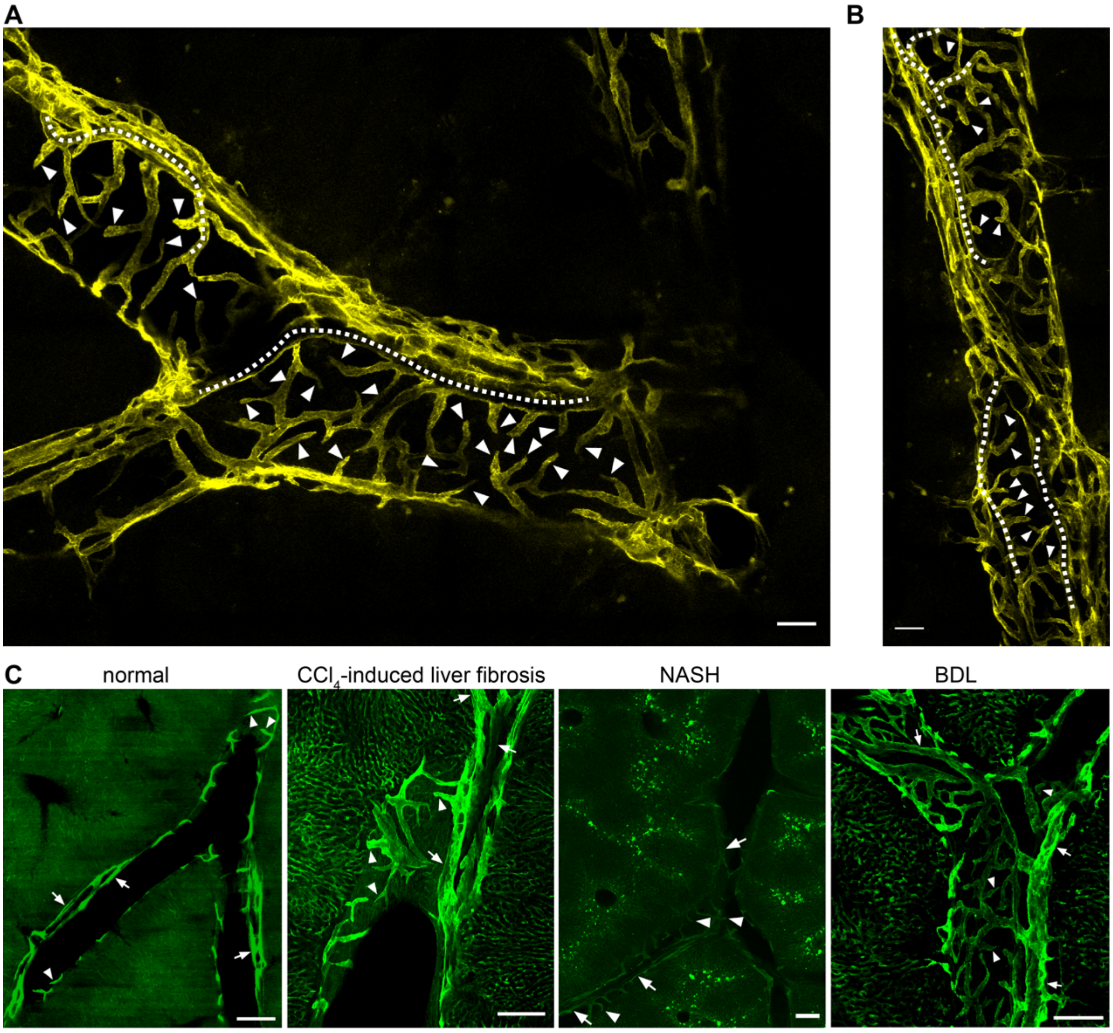

Figure S5. Three-dimensional mapping of hepatic LVs displayed the continuous structure of the lymphatic capillaries to the large LVs. (A-B) 3D imaging of hepatic LVs in normal mice displayed the continuous structure of the lymphatic capillaries to the large LVs. The white

arrowheads indicate the blind ends of lymphatic capillaries, and the white dotted lines indicate the continuous structure and large LVs. (C) The diameters of 3 lymphatic capillaries and 3 large LVs in 4 portal vein areas (including 2 large [diameter 300-400 μm] and 2 small [diameter 50-100 μm] portal vein areas) were measured (n = 3 mice in every group). The white arrowheads indicate lymphatic capillaries, and the white arrows indicate large LVs. LVs, lymphatic vessels. Scale bar: 200 μm.

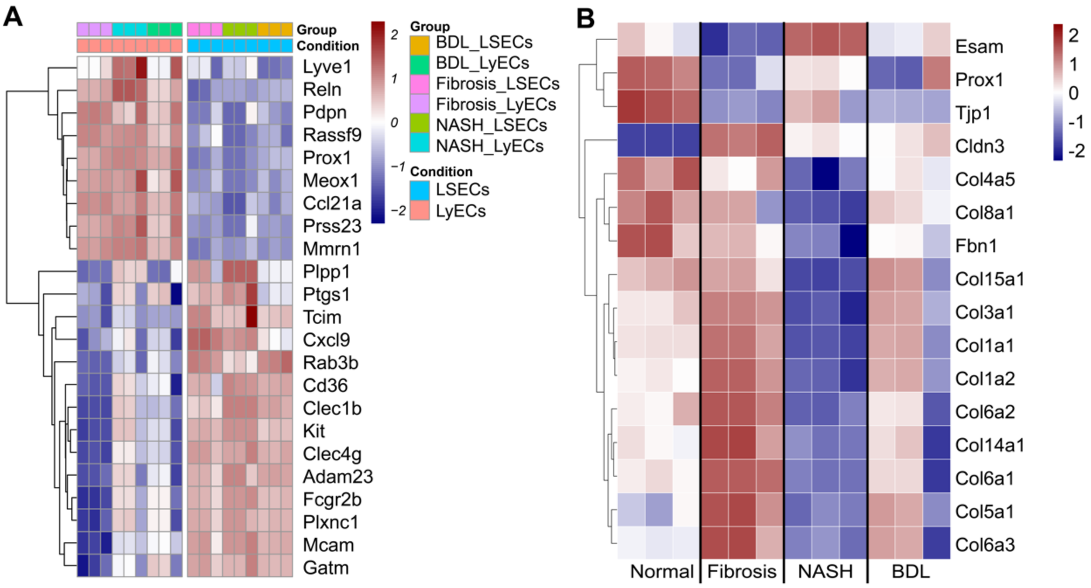

Figure S6. Transcriptome sequencing analysis and comparison of LyECs and LSECs between normal and diseased livers. (A) Heatmap with hierarchical cluster analysis of marker genes. The marker genes were selected from the DEG set of diseased hepatic LyECs compared to diseased LSECs. (n = 3 mice in each group). (B) Heatmap of selected genes of hepatic LyECs from normal and diseased livers (n = 3 mice in each group).

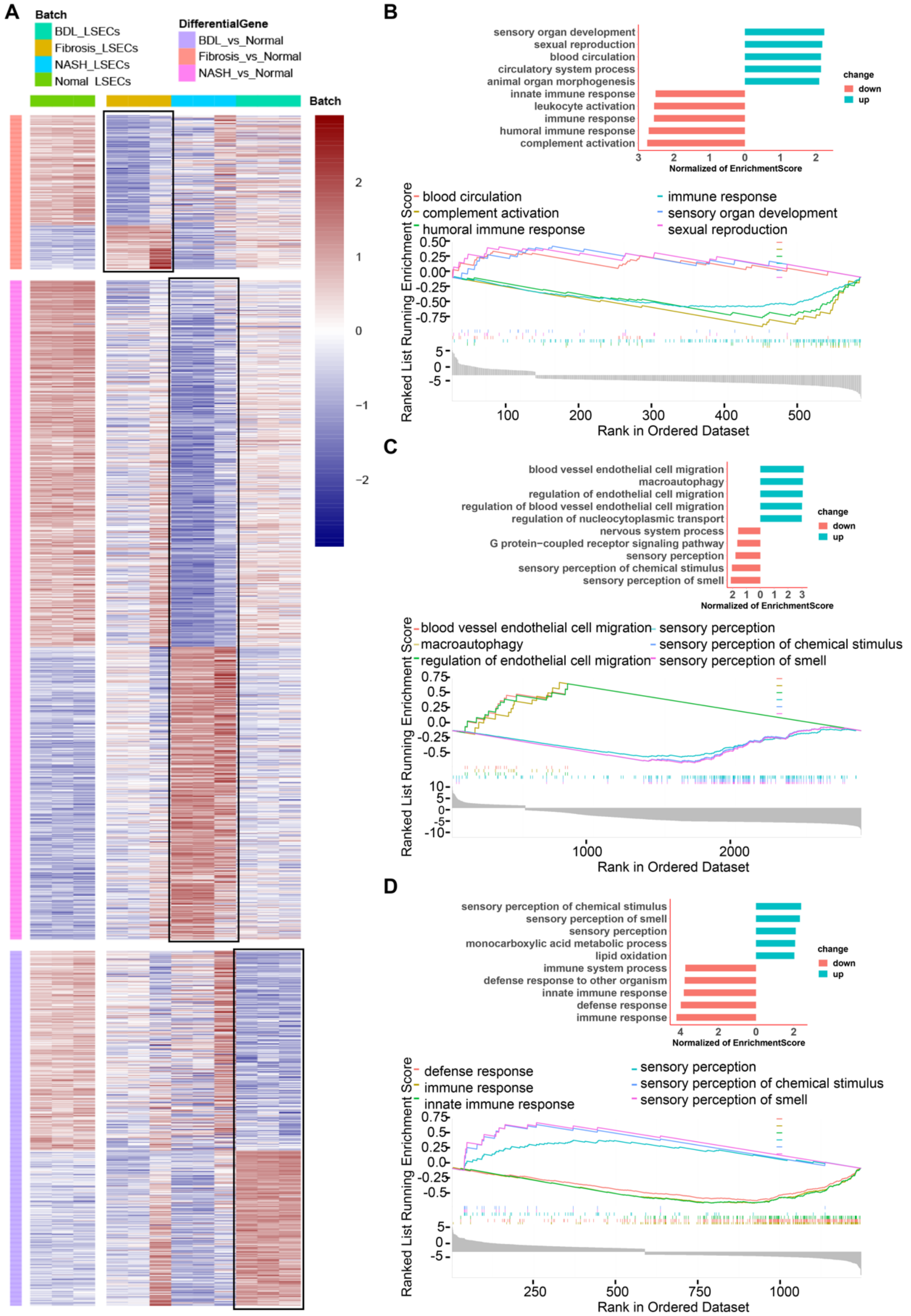

Figure S7. Transcriptome sequencing analysis and comparison of LSECs between normal and diseased livers. (A) Heatmap shows DEGs in LSECs between normal and three disease models in mice. (n = 3 mice in each group). (B) Gene set enrichment analysis (GSEA) for the LSECs from

normal and fibrotic livers using GO terms (top 10 terms). (C) Gene set enrichment analysis (GSEA) for the LSECs from normal and NASH mice using GO terms (top 10 terms). (D) Gene set enrichment analysis (GSEA) for the LSECs from normal and BDL mice using GO terms (top 10 terms). A P value < 0.05 and adjusted P value < 0.05 were considered significant; GO, Gene Ontology; NES, normalized enrichment score.

Other Supplementary Materials for this manuscript include the following:
Movies S1

Table S1 Catalog of antibodies used in this study.

Antibody	Clone	Company
Rat anti-mouse LYVE-1	ALY7	Invitrogen
Alexa Fluor 488-conjugated anti-mouse LYVE-1	ALY7	Invitrogen
Alexa Fluor 647-conjugated anti-mouse LYVE-1	223322	R&D System
Rat anti-mouse CD31	MEC13.3	BD Pharmingen
Alexa Fluor 594-conjugated anti -mouse CD31	390	BioLegend
Alexa Fluor 647-conjugated anti -mouse CD31	390	BioLegend
Goat anti-mouse CCL21		R&D System
Goat anti-mouse VEGFR3		R&D System
Syrian hamster anti-mouse PDPN	RTD4E10	Abcam
Syrian hamster anti-mouse PDPN	8.1.1	BioLegend
Rabbit anti-mouse Prox1	EPR19273	Abcam
Rabbit anti-mouse CK19	EP1580Y	Abcam
Brilliant Violet 421-conjugated anti-mouse CD45	30-F11	BioLegend
APC-conjugated anti-mouse CD146	ME-9F1	BioLegend
Alexa Fluor 488-conjugated donkey anti-rat IgG		Invitrogen
Alexa Fluor 568-conjugated goat anti-Syrian hamster IgG		Abcam
Alexa Fluor 568-conjugated goat anti-rabbit IgG		Invitrogen
Alexa Fluor 568-conjugated goat anti-rabbit IgG		Invitrogen
Alexa-Fluor 647 conjugated donkey anti-goat IgG		Invitrogen
Alexa-Fluor 647 conjugated goat anti-rabbit IgG		Invitrogen
